# Building youth power and environmental health literacy with environmental justice communities in rural Arizona

**DOI:** 10.3389/fpubh.2026.1733720

**Published:** 2026-05-12

**Authors:** Kunal Palawat, William Borkan, Sanlyn Buxner, Isabella M. Castañeda, Sallie Choi, Ted Choi, God’sgift N. Chukwuonye, Melissa Jaquez, Miriam Jones, Anastasia Mariscal, Miracle Martinez, Spencer T. McBride, Carol Newbauer, Caleb Ochoa, Benjamin Quesada, Maricela Quesada, Raquel N. Quesada, Iliana A. Samorano, Felix L. Vincent, Abigail Zettlemoyer, Mónica D. Ramírez-Andreotta

**Affiliations:** 1 Department of Environmental Science, College of Agriculture, Life, and Environmental Sciences, University of Arizona, Tucson, AZ, United States; 2 College of Education, University of Arizona, Tucson, AZ, United States; 3 Youth Advisory Board, “STEAM in Action”, Tucson, AZ, United States; 4 Regenerating Sonora, Inc., Superior, AZ, United States; 5 Mel and Enid Zuckerman College of Public Health, University of Arizona, Tucson, AZ, United States

**Keywords:** community based participatory research, community science, critical consciousness, environmental health literacy, environmental justice, Photovoice, rural, youth power

## Abstract

**Introduction:**

Resource extraction poses a substantial environmental justice problem globally. Community-based efforts to increase environmental health literacy have been linked with reducing exposure and increasing positive health outcomes. However, there is a gap in community-based environmental justice work centering around the education and needs of youth in rural communities.

**Methods:**

Using frameworks of environmental health literacy, an Ecological Model of Health, and Funds of Knowledge, we facilitated the ongoing STEAM in Action project, where we hosted two community-based youth environmental health trainings in environmental justice communities in rural Arizona. Photovoice, poster activities, environmental monitoring, data visualization, participant observation, pre- and post-surveys, focus groups, and follow-up interviews were quantitatively and qualitatively analyzed to understand youth environmental health literacy, concerns, and capacity for action.

**Results:**

Survey results indicated that 83% (*n* = 36) of youth’s motivation to take action increased as a result of the training. Environmental health literacy was demonstrated by youth’s knowledge and awareness of local environmental issues and solutions, highlighting environmental pollution from littering and industry, health issues like drug use, and a culture of stubbornness. Unique activities like Photovoice, environmental monitoring, and data visualization provided youth hands-on experiences to increase environmental health literacy.

**Discussion:**

Trainings through the “STEAM in Action” project allowed youth to surface taboo issues in their towns, while building the knowledge and skills to understand and address environmental justice issues in their communities. Future work will build out advisory boards, critical consciousness, and environmental justice trainings in service of collective action to affect community change.

## 1 Introduction

Resource extraction, stemming from colonial land relations, poses a significant global threat to human health (1–4). According to the US Environmental Protection Agency’s Toxic Release Inventory, metal mining is the largest producer of waste to the environment compared to any other industry (5, 6). Studies show that 90% of mined materials become polluting waste products (7, 8). This poses a major environmental public health concern, especially for marginalized communities who are at the frontlines of settler-colonial, capitalist harm (2, 3, 9–11). Environmental Justice (EJ) literature shows that communities can take action to pressure governments and corporations and create healthier lives for themselves (12–15).

Core to EJ action is a community’s knowledge about their environment and solutions to the issues they face. Environmental health literacy (EHL) is a person’s understanding of how hazards can affect their health. EHL is more than just knowledge and awareness; it includes one’s self-efficacy, defined as confidence in one’s ability and capacity to act on this knowledge to protect their health and change their community (16–18). An Ecological Model of Health (EMH) helps one to conceptualize interventions to EJ issues at multiple nested levels emphasizing the importance of people’s interactions with both their physical and sociocultural environments on the intrapersonal, interpersonal, institutional, community, and public policy levels (19). An updated version of the EMH adds culture as the largest concentric circle acknowledging social determinants of health (20).

EHL is necessary for youth in EJ communities because in a settler-colonial society, youth are under-served and under-resourced. They have little economic, social, or political power to advocate for their needs and opinions (21, 22). The disempowerment and oppression of youth is sometimes termed adultism, defined as the practices, systems, and cultures that subordinate youth and deny them agency, particularly marginalized youth (23–25). The underestimation of youth by adults is pervasive around the globe, a result of capitalist, settler-colonial, anti-Black, white supremacist culture, even in programs where adults strive to be allies to young people (21, 23, 26–29). In the environmental field specifically, youth, particularly Black, Indigenous, and brown youth of color, are often seen as disposable and their ideas and calls to action are frequently silenced (28, 30, 31).

A 2019 scoping review of health research related to resource extraction observed that out of 2,797 articles and book sections published in English from 1995 to 2015 from around the globe, only 4.6% were about children/youth (32). A 2013 review observed that only 15% (*N* = 399) of community-based participatory research projects published in English *actually* partnered with youth (33). The disconnect between youth and adults is not only detrimental to achieving environmental health justice (22, 34). Concomitantly, young people are a vulnerable population and sensitive to environmental health risks from resource extraction and colonialism (35, 36). Social and political identities exacerbate the issues youth face. Rural youth, youth of color, and low-income youth experience unique issues that are further marginalized (26, 30). However, young people have high levels of sociopolitical and experiential knowledge and capacity to make global EJ efforts more just, transformative, efficient, and legitimate (22). Youth must be supported to learn and engage in EHL and action.

Funds of Knowledge (FoK) is a framework developed to validate the knowledge that people of color have from their families that is not valued by the traditional Western school system (37). The FoK uplifts cultural knowledges and skills that are essential for families and individuals to function in society (37–40). Using critical race theory and cultural capital frameworks, Yosso expanded FoK beyond teachers and families into communities and describes the Community Cultural Wealth model (39, 41). This model highlights the different forms of capital or knowledge someone coming from a unique culture has, particularly for communities of color resisting white supremacy (41). The aspects of Community Cultural Wealth are linguistic, aspirational, familial, social, navigational, and resistant capital, which we use to partially frame community power in rural EJ communities (41).

Critical consciousness is the capacity for marginalized individuals to understand and act on social injustices via the reciprocal processes of critical reflection, political efficacy, and critical action (34, 42, 43). Critical reflection is one’s understanding and analysis of social inequities and/or structural oppression, including gender, race, class, age, and sovereignty. Political efficacy is one’s perception of their own and community’s ability and motivation to make change happen at multiple scales. Critical action is any activity and/or behavior that individuals or groups engage in to challenge social inequities, including direct action like protest and civic engagement like community building, with an explicit understanding of structural oppression (43).

**Figure 1:**
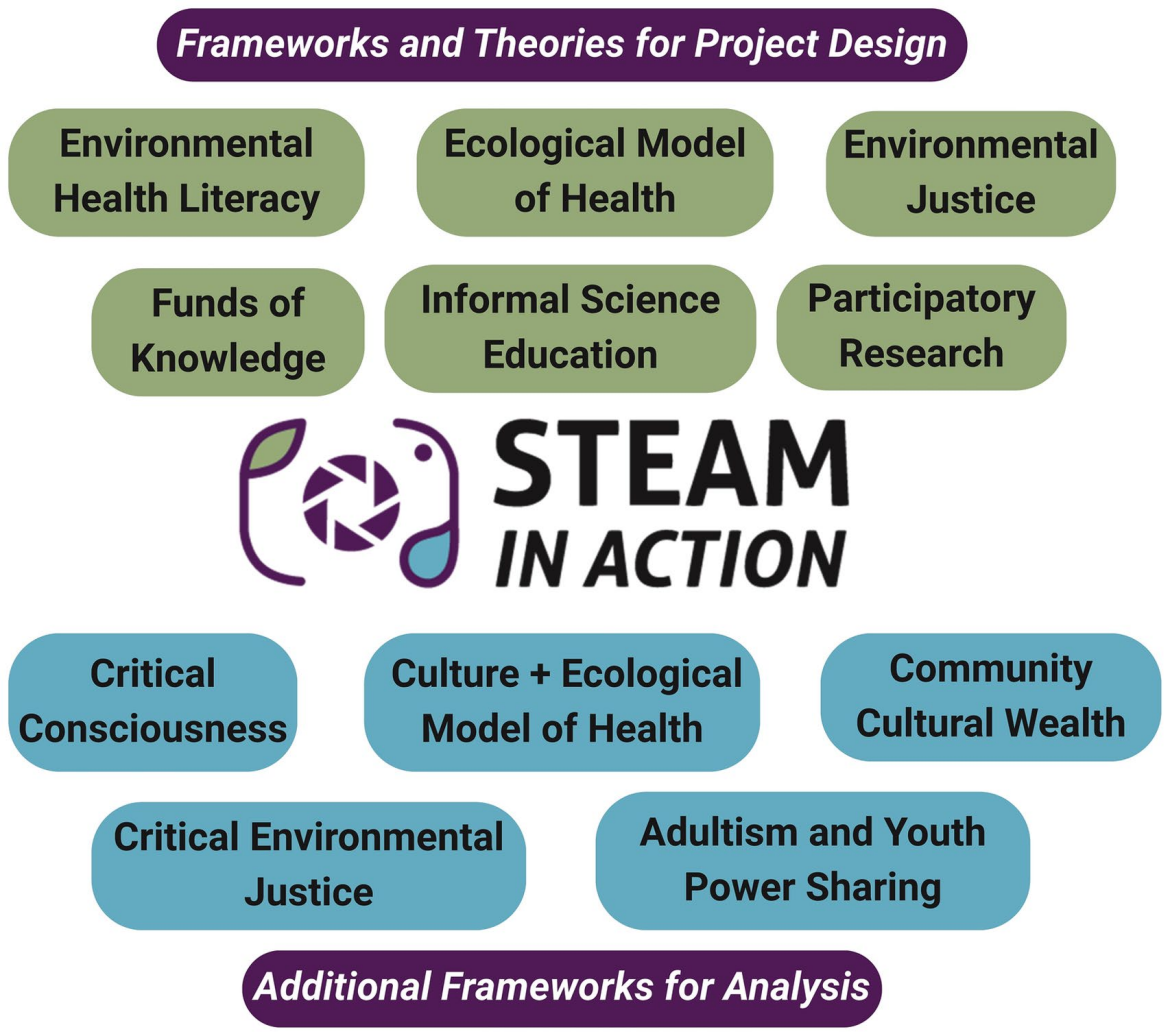
Frameworks and theories used in the creation and analysis of the “STEAM in Action” project.

Young people are at the forefront of movements for liberation and social change globally (22, 27, 44). Our work aims to support rural youth EJ efforts, support a culture of hope and action, and disrupt dominant narratives that victimize young people in EJ communities (22, 34, 45). This study reports on the results of “STEAM in Action”, a community-based participatory research project facilitating informal science education (46) research, grounded in frameworks of EHL, EMH, EJ, and FoK (Figure 1). Frameworks of Community Cultural Wealth and critical consciousness support the analysis and discussion (Figure 1).

## 2 Methods

### 2.1 Project overview and partnership building

This project builds on over a decade of community and citizen science efforts supporting environmental health justice in Arizona’s rural, active/legacy mining communities (47–55). The communities we worked with are considered EJ communities, disproportionately impacted by environmental harms; for a detailed description of these communities, see Davis et al. and Ramírez-Andreotta et al. “STEAM in Action” was developed in response to local adult community scientists/participants requesting hands-on science education for youth in the area. The goal of “STEAM in Action” is to create and pilot a national model of STEAM education that engages adults and 7th–12th graders to provide them with the tools, skills and support to document, reflect upon, and communicate issues of concern (through advisory boards and Photovoice), collect environmental samples (through co-created citizen/community science), and make sense of data so that their results can inform actions at the local, regional, and even national level (through data visualization and environmental communication).^1^

In 2023, the first year of the program, listening sessions (*N* = 2) and a teacher survey were administered in Pinal and Gila, AZ counties. In general, these efforts revealed teacher dissatisfaction with current science curricula/resources in schools and identified priority content areas for the STEAM in Action trainings. Based on the outcomes of these efforts, the Environmental Science, Health, and the Public Sphere trainings for both youth^2^ and adults^3^ were developed. Here, we describe the outcomes of the summer 2024 youth trainings.

### 2.2 STEAM in action youth trainings

As a part of a variety of educational efforts, in June 2024, we hosted two youth trainings in English for 7th through 12th graders in partnership with teachers, administrators, schools, community-based organizations, and community champions in Globe/Miami (GM) and Hayden/Winkelman (HW). As one way of valuing youth’s time and expertise, youth participants were compensated, with additional compensation for completion of certain evaluation tools. These trainings aimed to support EHL, community action, and youth empowerment in rural EJ communities. The facilitation team comprised of professors, promotoras (community health workers) (56), graduate and undergraduate students, and community partners. These trainings are micro-credentialed (verified, validated, and attested that the specific skills and/or competencies have been achieved), endorsed by University of Arizona, aligned with state and/or national science standards and include the following sections: Environmental Science and Health, Environmental Biology, Research Methodologies, Soil and Water Quality and Atmospheric Dust, Food Sovereignty, and Environmental Communication and Justice. See more at https://steamaction.arizona.edu/. Table 1 shows our aims and objectives for the trainings overall.

**Table 1:** STEAM in Action training aims and objectives mapping to evaluation frameworks and tools.

Aims and objectives	Evaluation frameworks							Evaluation tool
	Environmental health literacy	Ecological model of health	Environmental justice	Critical consciousness	Funds of knowledge	Community cultural wealth	Adultism and power sharing	
Participation in the Summer program will enhance participants’ understanding of environmental justice and its significance in their local community. Specifically, participants will be able to								
*1. Recall key public health and environmental justice definitions, activities, and moments in time.*	X	X	X					Pre- post-survey; Environmental science and ecological model of health posters
*2. Summarize and translate the state of public health and environmental justice and the associated controversies in their community.*	X	X	X	X	X	X	X	Pre- post-survey; Environmental science and ecological model of health posters; Photovoice; Semi-structured focus group; Follow-up interviews; Youth co-authorship
*3. Assess and appraise the sources of impacts to land resources in their community.*	X	X	X	X				Environmental monitoring and data visualization; Photovoice; Semi-structured focus groups
*4. Illustrate an understanding of the relations between environmental quality, justice, and human health.*	X	X	X	X				Environmental science and ecological model of health posters; Participant observation; Semi-structured focus groups; Follow up interviews; Youth co-authorship
*5. Value environmental sustainability and ecosystems functions, services, and management.*		X			X	X		Environmental science and ecological model of health posters; Environmental monitoring and data visualization; Photovoice; Semi-structured focus groups
*6. Judge environmental laws and regulations relevant to resource extraction and redevelopment.*	X	X	X	X			X	Pre- post-survey; Environmental science and ecological model of health posters
*7. Interpret how racial/ethnic minorities, women, LGBTQIA+ people, being disabled, and/or being from marginalized communities effects the environmental health of individuals and communities.*	X	X	X	X	X	X	X	Participant observation; Semi-structured focus group; Youth co-authorship
Capturing local perspectives through Photovoice will result in the following outcomes								
*1. When compared to traditional formal education methods, students will more deeply reflect and record their concerns relating to environmental quality and health.*	X				X	X		Photovoice
*2. Based on the state of the mine (*i.e.*, proposed, active, closed), politics, and community ecology, individual student’s concerns and communication strategies will differ.*			X	X	X	X	X	
*3. Photovoice will serve as an effective and positive vehicle for integrating history and healing practices into their community.*		X		X	X	X	X	
Participation in the co-created citizen/community science youth program will enhance students’ overall environmental health literacy, including								
*1. Understanding the scientific method.*	X							Environmental monitoring and data visualization; Youth co-authorship
*2. Understanding techniques and processes for environmental monitoring.*	X							Pre- post-survey; Environmental monitoring and data visualization
*3. Skills related to using soil and air monitoring instruments and following soil and air monitoring protocols.*	X							Environmental monitoring and data visualization
*4. Self-efficacy for learning and doing science, particularly science related to exposure mitigation.*	X	X	X	X	X	X	X	Pre- post-survey; Environmental monitoring and data visualization; Semi-structured focus group
Overall program impact goals								
*1. Acquisition of new environmental health and data literacy skills*	X	X						Pre- post-survey; Environmental science and ecological model of health posters; Semi-structured focus group; Follow-up interviews; Youth co-authorship
*2. Increased motivation to learn science, specifically environmental health science*	X				X	X		Pre- post-survey; Participant observation
*3. A shift of their view of science and scientists, specifically environmental science/health science*	X		X	X				Participant observation; Semi-structured focus group; Follow-up interviews; Youth co-authorship
*4. Interest in taking more science or government classes*	X				X	X		Pre- post-survey; Participant observation; Semi-structured focus groups
*5. Interest in participating in the debate team or other extracurricular activities that relate to environment, communications, and policy*	X				X	X		Pre- post-survey; Participant observation; Semi-structured focus groups
*6. Interest in STEAM careers*	X				X	X		Pre- post-survey; Participant observation; Semi-structured focus groups

A total of 49 youth participated in the trainings, with 42 youth completing 75% or more and receiving micro-credentials. The trainings were held in Hayden (*n* = 29) and Globe (*n* = 20), AZ, and had participants from surrounding Arizona towns, e.g., Winkelman, Kearny, San Manuel, Superior, and Miami. Youth were recruited via direct outreach by promotoras (e.g., information tables at community events, social media posts, telephone calls to local organizations and community members), teacher referrals, and by existing relationships between research staff and parents. The ethnic demographic of our youth participants—67% Hispanic or Latinx of any race (Table 2) falls within the range of Hayden, Winkelman, Globe, and Miami town demographics reported in Ramírez-Andreotta et al., 2023–16 to 76% Hispanic or Latinx of any race (53). Overall, 22% of our youth participants spoke Spanish, whereas the range in their communities was 15–62% (47), potentially highlighting a gap between youth and adult Spanish proficiency. Generally, our results are representative of youth experiences in rural AZ environmental justice communities, but communities with different sociodemographic, political, and ecological compositions may have uniquely different experiences. To leverage the momentum of the trainings, two STEAM in Action Youth Advisory Boards (YAB) were established in August 2024 in Hayden and Globe. In general, YAB members meet monthly and are paid to build community awareness, guide STEAM in Action education/research efforts, and inform data report back practices.

**Table 2:** Youth training demographic summary.

Demographic data						
Age (*n* = 36)						
10	12	13	14	15	16	17
3%	17%	28%	28%	14%	8%	3%
Grade Group (*n* = 36)						
7th and 8th	9th and 10th	11th and 12th				
56%	31%	14%				
Household Size (*n* = 34)						
3	4	5	6	7	9	
9%	18%	29%	21%	18%	6%	
Race and ethnicity (*n* = 30)						
American Indian or Alaskan-Native	American Indian or Alaskan-Native and Asian American, Asian or Pacific Islander	American Indian or Alaskan-Native and White or European	Asian American, Asian or Pacific Islander and Hispanic or Latinx	Hispanic or Latinx	Hispanic or Latinx and White or European	White or European
7%	3%	3%	3%	43%	20%	20%
Ethnicity (*n* = 30)						
Hispanic or Latinx	Not Hispanic or Latinx					
67%	33%					
*Person of Color (*n* = 30)						
No	Yes					
40%	60%					
Primary Language (*n* = 36)						
English	Spanish					
97%	3%					
Secondary Language (*n* = 36)						
English	Spanish	Japanese	Russian	I do not speak another language		
3%	19%	3%	3%	72%		
Training location (*n* = 49)						
Globe/Miami	Hayden/Winkelman					
41%	59%					
**Gender (*n* = 36)						
Man	Transgender	Woman				
56%	3%	42%				
Lived in community 5 + years (*n* = 36)						
No	Yes					
14%	86%					

*Based on their responses for race and ethnicity, respondents were categorized as a person of color if they selected any non-‘White or European” identity or not a person of color if they selected only ‘White or European’. **While the survey options included ‘Man’ and ‘Woman’, participants are referred to as boys and girls throughout the manuscript when referencing gender because of their age.

The study protocol for research was approved by the University of Arizona Institutional Review Board (IRB Protocol ID: 1507953512). Before any form of data collection, parents reviewed/signed a consent form for their child(ren) and youth reviewed/signed an assent form. To protect youth anonymity, some quotes in this article are attributed to a training community (HW or GM) and some are not. No youth names are used throughout the paper and all data have been de-identified.

### 2.3 STEAM in action evaluation tools

#### 2.3.1 Pre- and post-survey

A pre- and post-survey was designed to determine participant learning and perspective changes over the course of the training. The pre-survey also included a voluntary demographic survey. The pre-survey was administered before the training began, and the post-survey was administered at the end of the training (see survey questions in Supplementary file 1). Two participants started the training on the third day and took the pre-survey that morning. One participant took the pre-survey after 1.5 days of programming, and one participant took it after attending 2 days of programming. Pre- and post-survey data were matched, omitting surveys that had only pre or post completed—19 from Hayden/Winkelman (73%) and 17 from Globe/Miami (85%).

#### 2.3.2 Environmental science and ecological model of health posters

During the training, a key activity was for groups to select an environmental health challenge, research the topic, and develop a poster with the following sections: topic, source, human health connections, EJ connections, solutions, and needed or existing governance structures. Later in the training, groups took their selected environmental health challenge and were prompted to describe interventions at multiple scales. This activity used the EMH as a core framework, with interventions at the intrapersonal, interpersonal, institutional/organizational, community, and public policy levels (19, 57). A total of 46 youth participated in the two-part poster activity. See Supplementary files 2, 3 for complete lesson plans. Posters were transcribed by the research team for analysis.

#### 2.3.3 Environmental monitoring and data visualization

Youth were trained and asked to collect soil, dust, and water samples from their homes and analyze them using lab-validated Do-It-Together (DIT) methods, assisted by program staff. Youth were then tasked with documenting, interpreting, visualizing, and presenting the results in groups during the training. We held “Data Discos,” where youth shared their visualizations with the group and were celebrated for their work and creativity.

#### 2.3.4 Photovoice

The Photovoice activity was integrated into the learning research to explore how photography, reflection, and writing can be combined to record unique environmental health perspectives (58, 59). Participants were invited to consider what they learned through the training, take up to 5 photos highlighting something they care about or want to improve, then write a caption to accompany each photo. To prepare for the Photovoice activity, participants were encouraged to consider what it would be like to share their work with decision makers. *What do you want them to learn from your photos? What important issues do you want to talk about?* On the last day of each training, the participants Photovoice submissions were shared at the final celebration with everyone who took part in the training and with their families.

#### 2.3.5 Participant observation

Throughout the training program staff took notes on any relevant interactions, tensions, and behaviors during most lessons/activities (60). Staff also facilitated daily self and group reflections among the facilitation team to prepare for teaching, unpack the events of the day, and check in about the needs of individual youth. These data were used as reference material to ground-truth results and discussion points.

#### 2.3.6 Semi-structured focus groups

At the end of each training, semi-structured focus groups were conducted with youth participants to understand their training experience, how they were contextualizing the environmental health information, and potential learning outcomes. See Supplementary file 4 for the focus group script. In HW 23 of 29 youth participated in focus groups and in GM 17 of 20 youth participated; youth were divided into two focus groups at each training, totaling 4 focus groups. Focus groups took approximately 40 min, were recorded by the research team, and transcribed by Ubiqus (61). Focus group data were excluded from quantitative analysis if a participant could not be identified by voice, but unidentified quotes were included in the qualitative analysis.

#### 2.3.7 Semi-structured follow-up interviews

Between 6 and 8 months after the youth trainings, 13 out of 42 (31%) youth participants, who completed 75% or more of the training, were randomly selected for a follow-up interview to determine environmental health knowledge, skills/knowledge acquisition/retention, participant satisfaction, communication efforts, and efficacy for social change. To understand participant experience comprehensively, equal weight was given to those who did and did not pursue advisory board positions, and each community was proportionately represented. There were 11 youth interviews total (7 advisory board members and 4 non-advisory board members); two youth non-advisory board participants did not respond to researchers within the follow-up interview window. See Supplementary file 5 for the interview script. Recordings of the interviews were transcribed by Ubiqus (61).

### 2.4 Youth co-authorship

In line with our community-based research methods, the FoK framework, and the Community Cultural Wealth framework, our program staff invited youth to be co-authors on this paper. Staff wanted to formally honor young people’s expertise and contributions to the data, analysis, and discussions. Instead of simply sending an academic manuscript to middle- and high-school aged youth to review, program staff developed a hands-on co-authorship workshop as a way of training young people in the academic writing process and supporting youth leadership. All 49 youth from the summer 2024 trainings and youth from YABs were invited to the workshop in August 2025. Twelve youth attended and participated in qualitative coding, data analysis, visualization, and discussions using a subset of the data analyzed in this paper. The youth’s coding and reflections were then used to confirm the program staff’s analysis and deepen the discussion section. Youth input primarily deepened the Photovoice analysis, understanding of critical consciousness and action, and understandings of adultism in rural communities. Additionally, some youth-designed visualizations were adapted for this manuscript. Ten of the young people opted to be named co-authors on this publication. All co-authors were also sent the manuscript draft for review before submission.

### 2.5 Evaluation framework and data analysis

A mixed-methods qualitative and quantitative approach allowed the research team to evaluate youths’ complex and nuanced understandings of environmental health, youth power, and environmental justice. See Figure 1 for a conceptual diagram of all our evaluation frameworks. Table 1 aligns evaluation frameworks and evaluation tools to specific project aims/objectives.

Quantitative, multiple-choice pre/post survey responses were analyzed by Chi^−^squared (Chi^2^) tests to assess for an association between survey type and response, with an alpha of 0.05. Likert-scale questions were analyzed by univariate and bivariate ANOVAs to determine significant differences by community and demographic variables See Supplementary Table 1 for detailed summaries of statistical models used in the analysis. Transgender participants were excluded from analyses of gender due to low n-values and to maintain anonymity. This means that the results are not necessarily generalizable to transgender youth, particularly our gender-focused analyses. And while the survey options included “Man” and “Woman,” participants are referred to as boys and girls throughout the manuscript when referencing gender because of their age. Based on their responses for race and ethnicity, respondents were categorized as a person of color if they selected any non-‘White or European’ identity or not a person of color if they selected only `White or European`.

Qualitative research methods allowed the research team to understand the training themes, aims, and objectives from a personal narrative perspective compared to quantitative evaluations. Validity and reliability of qualitative narratives were ensured by trust building between research team and youth participants and by an iterative and collaborative analysis process (53, 54). To organize and analyze the qualitative datasets, the research team developed a project codebook primarily via an inductive process, documenting and organizing emergent themes from each dataset. A deductive process was used to assess several project core themes, i.e., EHL (16), critical consciousness (43), and EMH (20). Transcripts were coded for codebook themes using NVivo 14 for Windows (62), which was then analyzed using Nvivo and R (63). Three researchers coded the data and met weekly to discuss the data and coding process. When a researcher was unsure about a code or if there was disagreement, the three researchers would discuss and re-code the segment together, updating and detailing the codebook as necessary. Codebook updates triggered researchers to re-code of all datasets to ensure thorough and equal analysis. The intercoder reliability was calculated using the Kappa coefficient on a random sampling of 15% of transcripts. Across all codes and chosen files, the analysis resulted in an average 87% percent agreement and average kappa coefficient of 0.56, which is considered fair to good (64).

## 3 Results

Thirty-six out of forty-nine training participants submitted demographic information. Table 2 shows the demographic breakdown overall and by community. Participant ages ranged from 10 to 17 years old, with 56% of youth being 13 or 14; 67% of respondents were Hispanic or Latinx; 60% were categorized as people of color; 56% identified as boys, 42% as girls, and 3% as transgender; and 86% of youth have lived in their community for 5 + years (Table 2).

Based on the frameworks provided in the introduction, the results section is organized to describe youth EHL, critical consciousness, and training reflections.

### 3.1 Environmental health literacy

#### 3.1.1 Awareness

**Figure 2:**
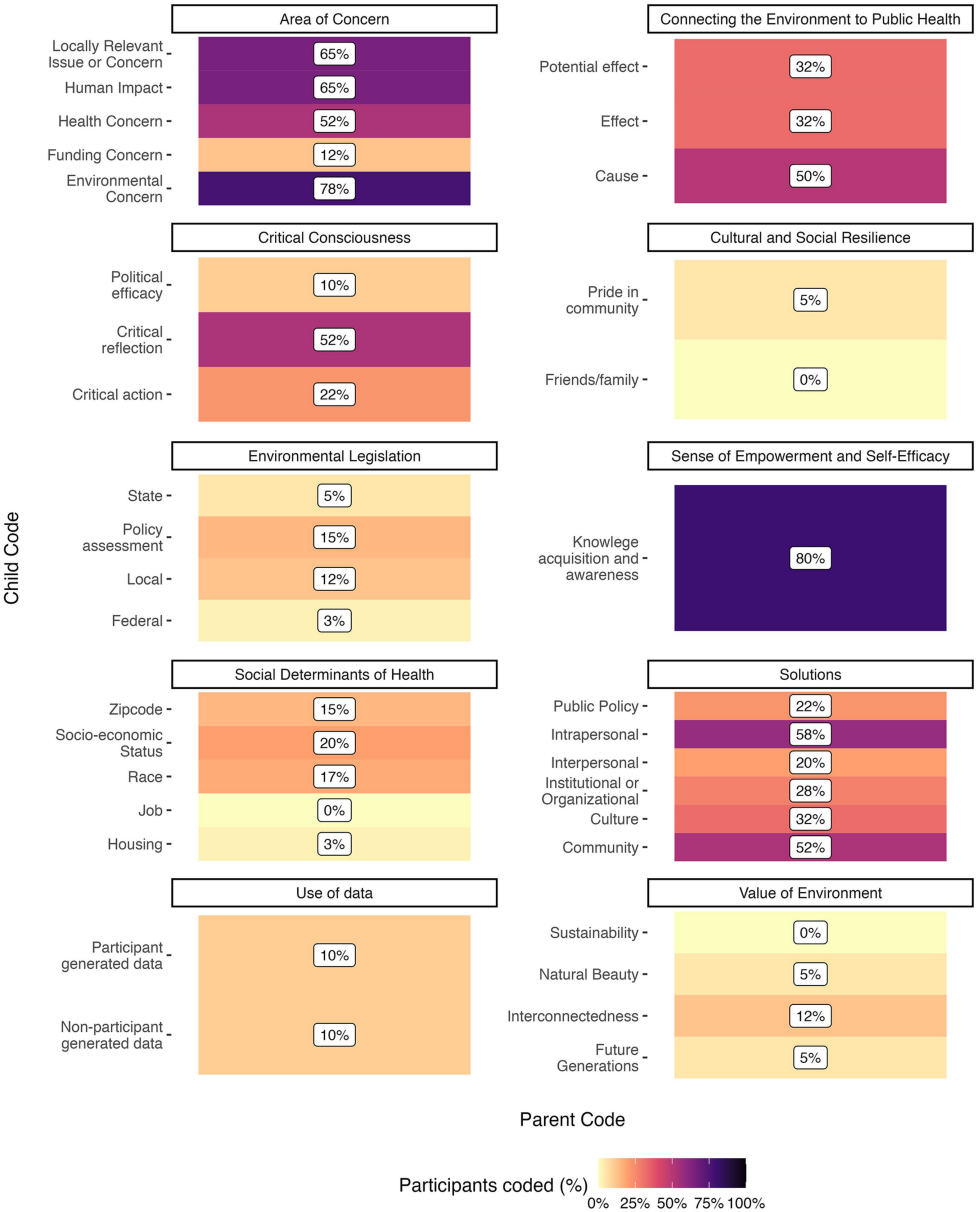
Heatmap showing the percent of focus group participants coded to parent and child codes. Values will not add up to 100% because child code values represent the percentage of total focus group participants who mentioned the child code (*N* = 40).

The youth participants value and place themselves in relationship with the environment. In focus groups, themes of interconnectedness, beauty, and future generations came up for 12 youth (*N* = 40; Figure 2). A youth in one focus group said,

*I think it’s important to care about the environment, no matter who you are […] The soil being bad affects our water and our plants. It’s really important to care about these things. You should not just be like, well, it’s fine here, so let us go over here, and not worry about that. I think it’s important to care about everywhere.*

**Figure 3:**
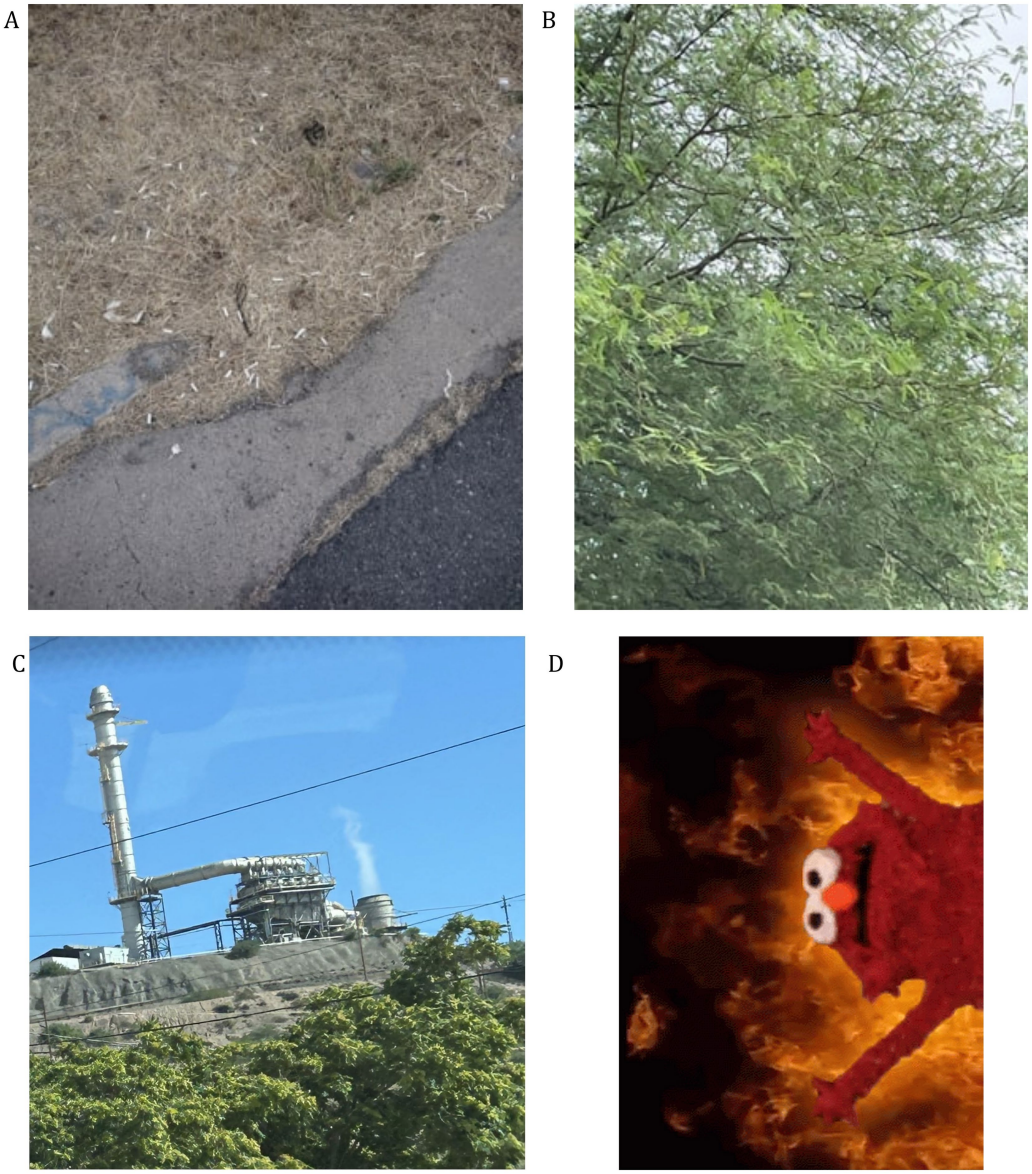
Youth photovoice submissions. **(A)** “…cigarettes are bad for humans so you got to think of how bad they are for animals if an animal ate them what would happen let’s not smoke for the environment.” **(B)** “Look at this tree, and noticed how it’s alive. When we take care of our environment and do our part we are able to have a better planet and ecosystem. With that we need to be able to take care of each other and Mother Earth.” **(C)** “Here is some air pollution I found locally while driving to the arboretum.” **(D)** “This meme reminds me of the chaos within forest fires and how we should try to prevent them”.

In Photovoice, 66 (55%) of 121 total submissions were coded to value of the environment, particularly regarding interconnectedness and sustainability. One youth’s submission interwove the value of the environment with socioecological health by featuring a photo of a tree, with the caption (Figure 3),

*Look at this tree, and noticed how it’s alive. When we take care of our environment and do our part we are able to have a better planet and ecosystem. With that we need to be able to take care of each other and Mother Earth.*

However, youth relationships with the environment were not all positive, as seen in other environmental health programs such as “Our Voices” (65) and “A Day in the Life” (66) youth awareness of environmental and justice issues increased as a result of the training. Based on pre- and post-survey data, the perception of environmental problems increased from 67 to 72% and the perception of injustice problems in the community increased from 11 to 28% respectively, but these increases were not significant based on chi^2^ tests (Supplementary Figures 1, 2; Table 3). The most notable increase in the perception of injustice/justice problems was in GM, which went from 8 to 35% of youth saying they saw problems in the community (Supplementary Figure 2). Similarly, across all communities, the percentage of Hispanic or Latinx youth who perceived injustice/justice problems increased from 8 to 35% (Supplementary Figure 2). Notably, none of the non-people of color saw injustice/justice problems in the community in the pre-survey, and 36% did in the post survey (Supplementary Figure 2). Higher grade levels typically saw a greater increase in perception of environmental and justice problems in the community than lower grades (Supplementary Figure 2).

**Table 3:** Quantitative pre/post survey analysis summary, highlighting significance (*p* < 0.05) and nearly significant (0.1 > *p* > 0.05).

Question	Statistical test	Overall pre- and post-survey	Socioeconomic/ demographic variable pre- and post-survey model results				
			Community	Gender	Ethnicity	Race	Grade group
How would you rate your understanding of what is done in environmental monitoring (testing samples from the environment to see if it is safe)	ANOVA	post score greater than pre; *p* < 0.001	--	--	--	--	--
How would you rate your ability to use tools and directions to test soil?	ANOVA	post score greater than pre; *p* < 0.001	--	--	--	--	--
How would you rate your ability to use tools and directions to test the air?	ANOVA	post score greater than pre; *p* < 0.001	--	Boys greater than Girls; *p* = 0.029	--	--	--
How would you rate your ability to use tools and directions to test dust?	ANOVA	post score greater than pre; *p* < 0.001	--	--	--	--	--
How confident are you in your ability to LEARN science about reducing your exposure to dangerous stuff in the environment?	ANOVA	post score greater than pre; *p* < 0.001	--	Boys greater than Girls; *p* = 0.033	--	--	11th and 12th greater than 9th and 10th greater than 7th and 8th; *p* = 0.022
How confident in are you in your ability to DO science about reducing your exposure to dangerous stuff in the environment?	ANOVA	post score greater than pre; *p* = 0.005	Globe/Miami greater than Hayden/Winkelman; *p* = 0.057	Boys greater than Girls; *p* = 0.091	--	--	11th and 12th greater than 9th and 10th greater than 7th and 8th; *p* = 0.040
Post-survey only: How satisfied are you with the STEAM in Action training?	ANOVA		--	Girls greater than Boys; *p* = 0.068	Hispanic or Latinx greater than Not Hispanic or Latinx; *p* = 0.020	--	--
Have you heard the term “environmental justice” before?	Chi^2^	More yes in post survey; *p*-value<0.001	NA	NA	NA	NA	NA
Have you heard the term “public health” before?	Chi^2^	More yes in post survey; *p*-value = 0.077	NA	NA	NA	NA	NA
Have you heard the term “environmental monitoring” before?	Chi^2^	More yes in post survey; *p*-value = 0.0074	NA	NA	NA	NA	NA
Do you think that there are environmental problems in your community?	Chi^2^	--	NA	NA	NA	NA	NA
Do you think that there are justice or injustice problems in your community?	Chi^2^	--	NA	NA	NA	NA	NA
Do you think that you and others in your community are able to help make decisions about having a safe environment to live in?	Chi^2^	--	NA	NA	NA	NA	NA
Do you think that you and others in your community are able to help make decisions about social justice issues (i.e., issues that are about the fair and equal treatment of everyone)?	Chi^2^	--	NA	NA	NA	NA	NA

Column C highlights the results of models comparing the difference of pre- and post-survey responses of the entire cohort of youth. Columns D–H highlight the results of pre and post models with the addition of a demographic variable.

Over half (54%) of the 121 youth Photovoice submissions illuminated personally relevant concerns. HW youth highlighted humans’ impact on the local environment (i.e., littering, carving on trees, smoking), extreme temperatures, and infrastructure concerns (i.e., abandoned buildings and lack of funding for schools; Figure 3; Supplementary Figure 3). GM youth primarily mentioned pollution, air pollutants, and litter/trash, and were less locally focused than HW submissions (Figure 3; Supplementary Figure 3).

When asked what issues felt most pertinent to them at the end of the training, 78% of focus group youth (*N* = 40) named an environmental concern and 65% named a human impact (Figure 2). The most frequently discussed specific environmental concerns were pollution (40%), litter/trash (35%), air/dust (25%), and abandoned buildings (22%; Supplementary Figure 4). This aligns with what the youth picked as topics for their poster activities during the training. Other environmental areas of concern were transportation and local infrastructure, water (including contamination and flooding), climate change and extreme weather, flora and fauna health, soil pollution, stray dogs, and fossil fuels (Supplementary Figure 4). The issues of community esthetic, openness, and safety were important to young people as well. A youth connected air quality and dust issues to abandoned buildings, stating,

*I think it’s just pretty dirty here. I do not know how to explain it. There’s a lot of abandoned buildings, and it’s very dusty. And whenever the dust picks up, it just collects more and then the dust goes up and it takes it out and pulls it out. And it always blows into where I live. So, it’s dirty. […] Yeah, the buildings, most of them are burned. But there’s dust inside and it’s like they can breathe it in and stuff and get sick.*

Health concerns were also important to youth, with 21 focus group participants naming emotional well-being, eco-anxiety, respiratory issues, cancer, and/or vaping and smoking (Figure 2; Supplementary Figure 4). In the focus group, someone said,

*And I’m worried about mainly how here in the West we get really hot temperatures, especially during the summer. We get 100 s, we go up to 110 on some days. And I’m worried because of that. And I feel like we know this issue a whole lot, but do not take too much to help prevent it. I’m worried most about drought and how rivers are instead of rising going down here in the West. And I’m just worried that eventually we could possibly lose those rivers and that could cause a whole bunch of problems like no water, and no fish in those rivers, and eventually, if other rivers drought to a point where they are gone, we could possibly lose fish here in the West.*

Both youth communities highlighted wildfires as a concern, with a youth specifically referencing the 2021 Telegraph Fire that bordered Globe, Arizona. Another youth said,

*The air pollution around here. We are surrounded by towns that frequently get lit on fire, whether that’s manmade or just from lightning strikes from our monsoon season that’s happening right now. But because we are in such a middle zone of all these places, all of the smoke just travels toward – into our town, therefore just having us breathe it in. And for some people, like asthma, that can actually kill them from inhaling so much smoke.*

*And the thing with the mines as well, all that stuff they just put into the air just because they can, because they are making money as a company.*

Twenty-six (65%) of 40 total focus group youth mentioned human impacts as an area of concern, including industrial activity and littering, which were coded using the EMH framework (20) (Figure 2; Supplementary Figure 4). Similarly, in follow-up interviews, 91% of 11 youth mentioned cultural areas of concern and 45% discussed institutional or organizational concerns. In a focus group, a youth said,

*Personally, I looked into the Globe water, and I found out that they are – there were tons of stories about chemical spills or stuff from the mines getting into our pipes. And these things can be like – it can take centuries to clean up. And I think our community should know about that, because from a study from 2019, there were 15 chemicals found in our water, which is extremely like argh! One of the highest ones being arsenic tested in our water, which is also argh. It’s extremely scary. That should be public information. I think we should be testing our water more, especially with all those mines around. And I think things like Freeport should be telling us where they waste – where they put their hazardous waste dumps. I’ve seen that there’s a hazardous waste dump in Globe, but I could not find any information on it, nor does Freeport or the other mine […].*

In a focus group, a youth said,

*I did not know they had chemical in certain things that we use, like makeup. I did not know half the things that were in water either. I was drinking water without knowing that too. That was surprising.*

Youth also placed themselves within the culture of the community and acknowledged some the collective challenges to EJ awareness. In a focus group, someone said,

*I feel like it’s because we cannot see chemicals. We cannot determine. We do not know. If there’s a fire we can see that, but with chemicals we cannot see chemicals, so it does not like, we just do not really care.*

#### 3.1.2 Knowledge

Throughout the training youth demonstrated knowledge about environmental issues through their use of vocabulary, definitions, examples of environmental health problems, and their use of data.

**Figure 4:**
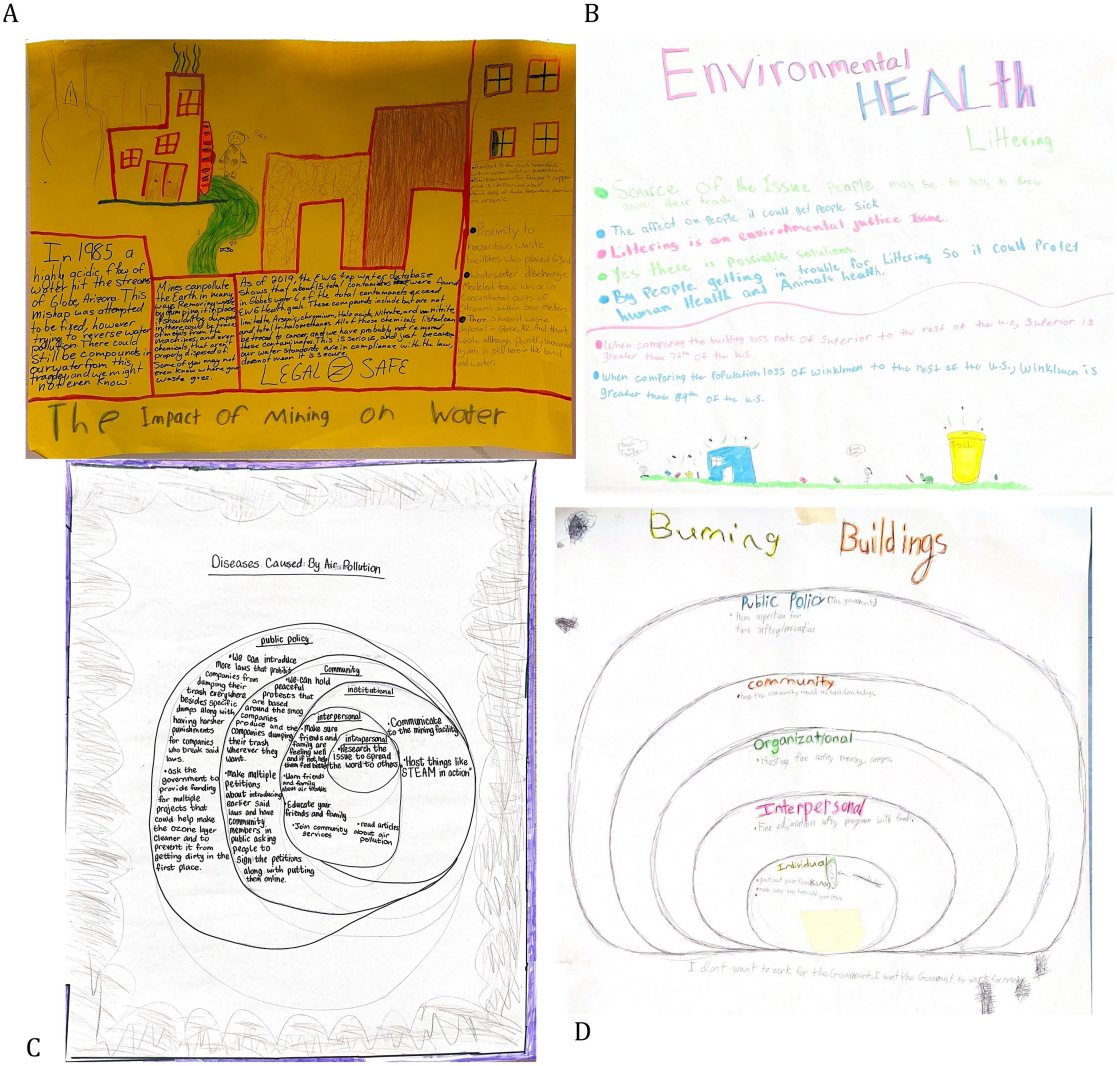
Images of youth introduction to environmental science **(A,B)** and ecological model of health posters **(C,D)**.

The introduction to environmental science, health, and policy posters, along with the EMH intervention posters allowed young people to research an environmental health issue of their choosing, primarily focusing on air pollution, water pollution, or trash (Figure 4). In their posters, with the support of program staff, youth wrote specific definitions for environmental health vocabulary words like wildfires, water pollution, and diseases such as pneumonia and strokes. They also exhibited understanding of environmental legislation by referencing laws discussed in the training, such as the Clean Air Act. A GM group went beyond referencing a policy and suggested improvements, writing, “just because our water standards are in compliance with the law, does not mean it is secure. LEGAL ≠ SAFE” on their poster (Figure 4). Youth also made direct environmental health and EJ connections by writing about factory worker occupational health, lower-income communities having fewer public services like cleaning and recycling, cyclists inhaling air pollution from cars, mines polluting local rivers, and wildfires exacerbating existing health disparities.

Familiarity with environmental laws/regulations also increased from pre- to post-surveys, with 72 to 86% of youth saying they were at least a little bit familiar or aware (Supplementary Figure 5). In the post-survey, six of those youth named specific laws and regulations such as “Companies can only produce a certain amount of pollution. Water can only have 10 ppb of arsenic*”*, “Well, there are tons of things in place, of course I can’t name any specifically, but there’s the EPA, WPA, all of the scientists that are apart of non-profits”, and “I know about the air pollution law that if companies put to much chemicals into our air they could get fined/reprimanded badly by federal law.” In focus groups, environmental legislation was briefly discussed, with most youth identifying issues at the local level (12%), then state (5%), then federal (3%; *N* = 40; Figure 2). Environmental laws and regulations did not emerge substantially in posters, Photovoice, or follow-up interviews.

**Figure 5:**
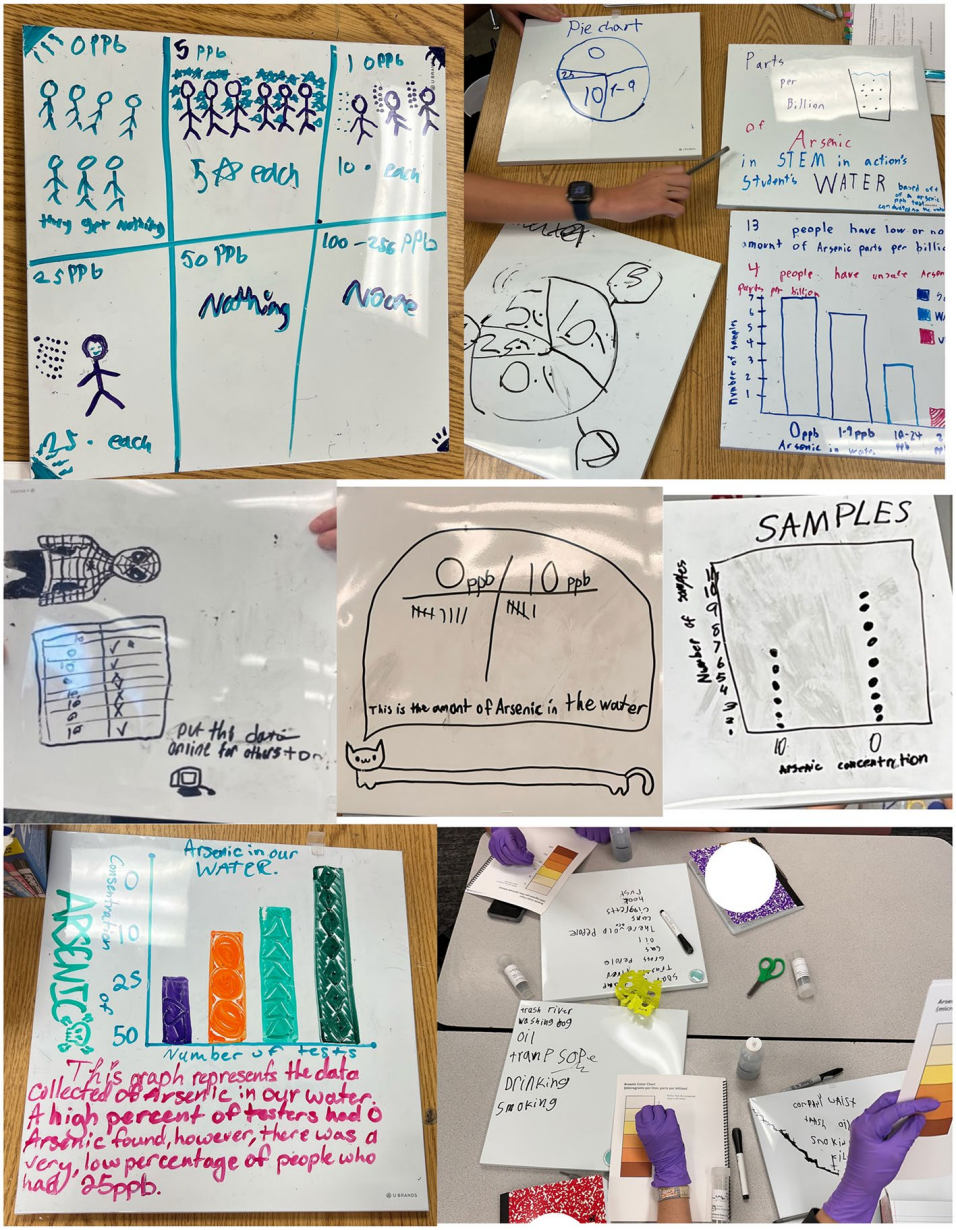
Images of youth conducting environmental monitoring, data analysis, data visualization, and sharing results during the summer 2024 trainings.

In their posters and discussions, some youth cited statistics from government websites and studies (Figure 4). Others used general comparison words like “more than” or “a lot”, particularly when discussing exposures to contaminants. Additionally, youth had the opportunity to analyze, visualize, and share their environmental monitoring results through “Data Discos.” They created tables, creative artworks, bar plots, and other graphs to help interpret and communicate their results with the larger group (Figure 5). Youth also created visualizations and communication strategies that were incorporated in the digital Photovoice explorer (available at: https://explore.steamaction.arizona.edu/Photovoice).

Photovoice submissions provided another outlet for using environmental vocabulary, with 61% of the 121 submissions containing an environmental vocabulary word (i.e., mining, pollution, soil, dust, emission, etc.). The GM youth’s vocabulary primarily centered around air pollution, with the use of words like “smog” and “ozone.” HW youth’s vocabulary centers around flora, fauna, and the weather. Almost a third of youth submissions demonstrated understandings of the environment’s connection to public health through the use of environmental vocabulary with narratives around wildfires, industries, and abandoned buildings. These results affirm the utility of Photovoice in building environmental health literacy as discussed in the literature (67, 68).

**Figure 6:**
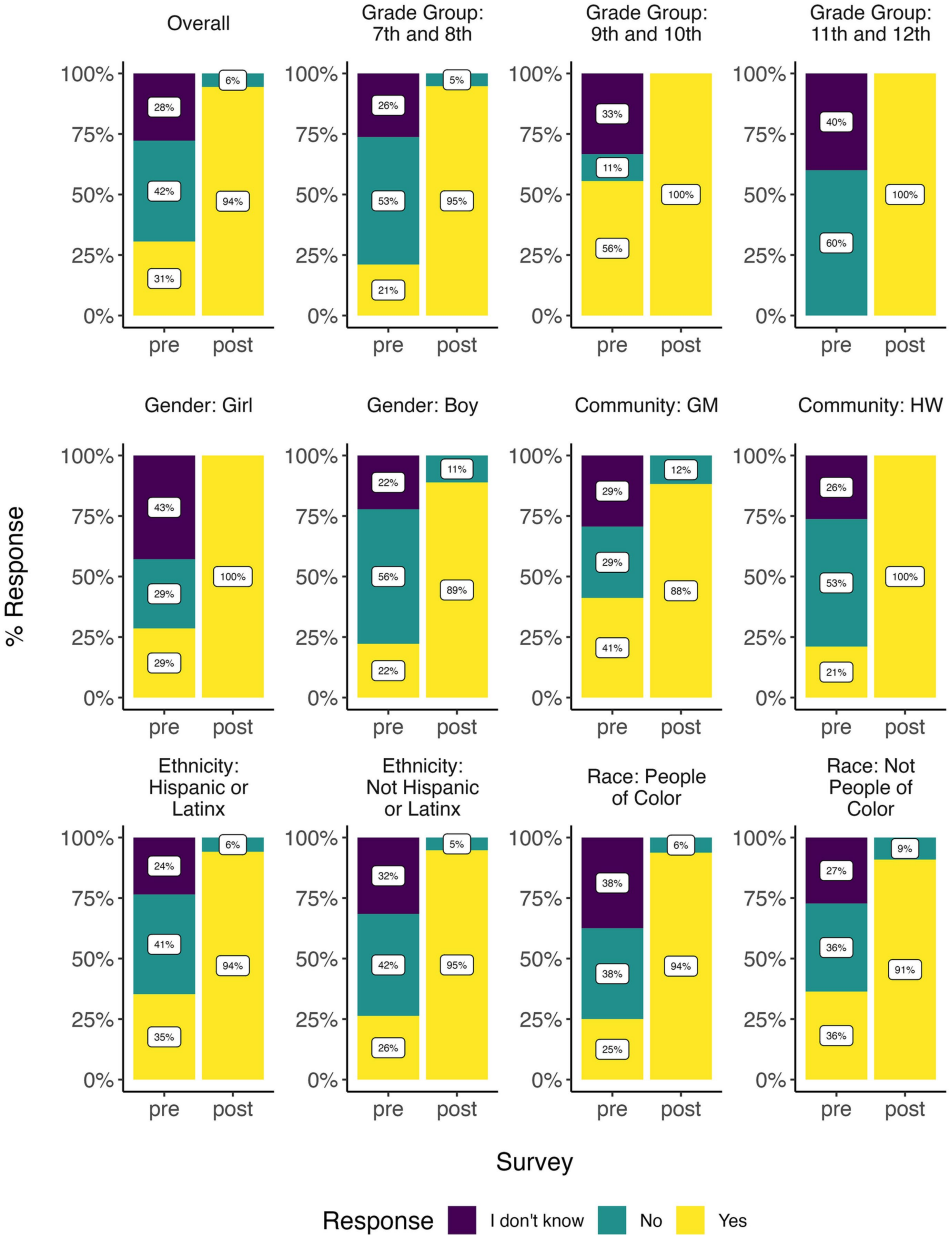
Bar plot summarizing pre- and post-survey results for youth who took both the pre and post surveys. Plots show responses to familiarity with the term, “environmental justice,” demonstrating environmental health literacy knowledge.

By the end of the training, youth were able to make numerous connections between environmental issues and health effects, especially relating to marginalized communities. Youth directly and indirectly discussed social determinants of health like race, Indigeneity, class, zip code, employment, gender, and housing in various training activities and artifacts. Based on survey data, before the training, 31% of youth were familiar with the term “environmental justice” (Figure 6) and 45% were able to correctly select the definition from a list (Supplementary Figure 6). After the training, significantly more youth (94%) were familiar with the term (*p* < 0.001) and 68% could correctly define it (Figure 6; Supplementary Figure 6; Table 3). A similar trend was observed with familiarity and definitions of the terms “public health issue” and “environmental monitoring” (Supplementary Figures 7–10; Table 3).

In a follow-up interview, when asked to define EJ, a youth said,

*It’s like red lining, so certain groups of people would get a better environment than other groups of people, like minorities, they would get red lined. They would get a worse environment. Maybe they had high levels of lead in their paint. Maybe they had air pollution. Maybe they did not have the best water, stuff like that, opposed to other, I cannot think of the word, or just like other groups of people who had better water, who had better air, who had better paint, who had just everything better.*

In discussing the disparity between the more racially diverse and lower-income community of Miami to Globe, AZ, a youth connected their lived experience to the traditional environmental justice distributive justice issue (69),

*[…] when we were learning about this stuff, I kind of got to see that, like how it is in our communities here, like how it’s treated, like whether it’s Globe, it’s Miami, and then how like in my neighborhood, it’s not respected as much, like how they enforce different policies and stuff. […] So, what that looks like in my neighborhood would probably be – is just we do not have like – like from the city being able to come up and clean our streets. We usually have to do it ourselves, like shoveling out when it floods, like rocks across our streets that are piled up, dirt, and then just like with littering, and like the abandoned houses there with trash. People just dump it onto other properties that they know aren’t going to get attended to.*

#### 3.1.3 Self-efficacy

The data also show that youth’s self-efficacy and motivation for learning/doing science, as well as decision-making increased due to the training. There were numerous indicators of a sense of empowerment, self-confidence, and trust in their own knowledge.

Youth expressed both excitement and nervousness about participating in environmental monitoring activities where they were trained and asked to collect and analyze soil, water, and air pollution data using a variety of lab-validated DIT methods. In focus groups, youth made statements indicating their increased capacity to affect change and do science. A youth appreciated the hands-on environmental monitoring skills that required certain technology and materials and discussed the importance of no-tech options, stating,

*I think it was all pretty helpful. Especially with the skill sets. That was a great learning experience for me but also probably everyone else. But I would say it should provide more what we could actually see and do. Like, with soils you have to dig to see that. But in like air you have to bring out all these monitoring devices and stuff like that. But I think something that we could actually do is identify things from the eye. Like littering or that water is bad or just things around us that people may not notice because they just glimpse over it.*

A youth demonstrated self-efficacy for making data-driven decisions saying,

*[…] Every time I’m probably going to do something with the water, I might be more cautious because of the test we did. Especially when we are doing the dishes, I’m gonna be more careful like not using as much and not like doing them for so long, like an hour long. I do not, because we found out there’s every ten billion, no, every ten droplets in a billion have that in it, so I might want to be more careful. Arsenic.*

**Figure 7:**
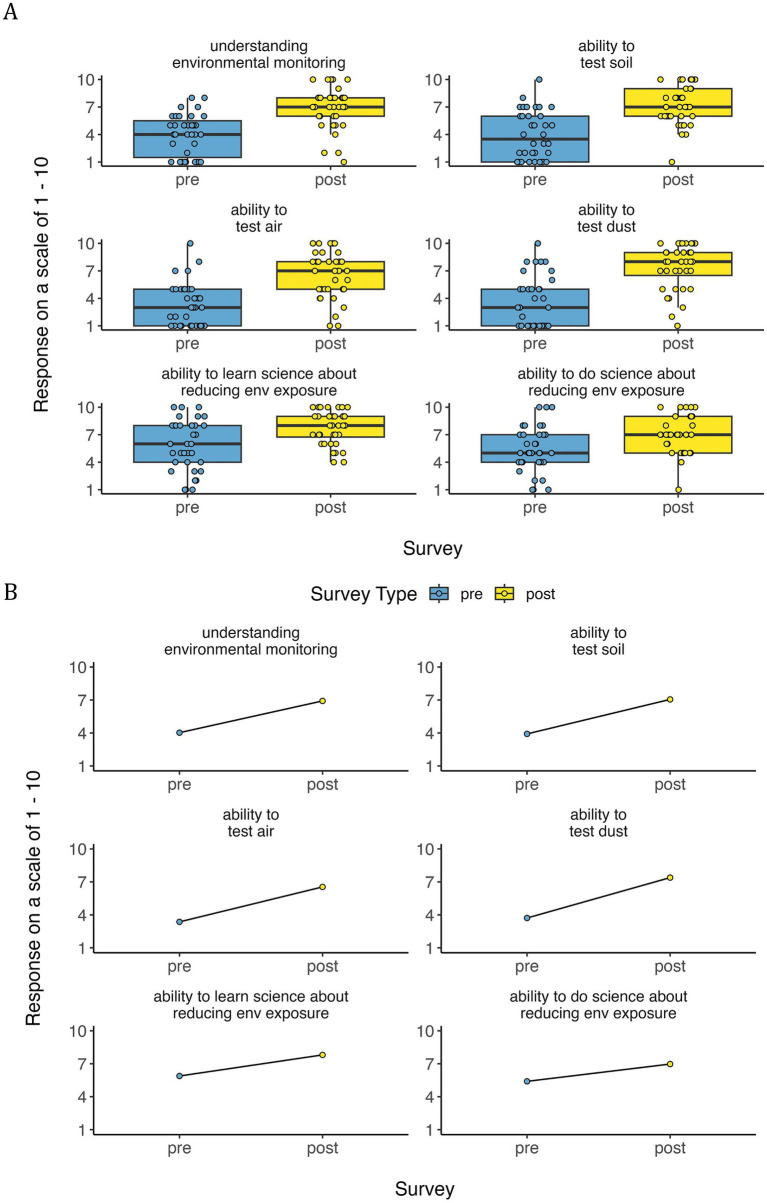
Youth self-rated confidence in learning and doing science, demonstrating environmental health literacy—self-efficacy through **(A)** boxplots and **(B)** line plots. Line plots showing mean values for pre- and post- surveys were designed by youth co-authors. Post survey values were significantly higher than pre survey values (Table 3).

Based on pre-and post-survey data, youth participant self-confidence in their ability to understand and conduct environmental monitoring and learning/doing pollutant exposure reduction significantly increased over the course of the training (*p*-values < 0.05; Figure 7; Table 3). This difference was most notable across grade levels, with higher grades generally showing greater increases in self-confidence than lower grades, a trend that was significant for both learning exposure science (*p* = 0.02) and doing exposure science (*p* = 0.04; Supplementary Figure 11; Table 3). Girls generally had lower self-confidence, significantly so for testing air (*p*-value = 0.03) and learning exposure science (*p*-value = 0.03) and nearly significant for doing exposure science (*p*-value = 0.09; Supplementary Figure 12; Table 3). However, both girls and boys showed similar increases in self-confidence, girls just typically had a lower baseline self-reported confidence than boys (Supplementary Figure 12). There were no significant differences by community, ethnicity, or race (Supplementary Figures 13–15; Table 3).

Furthermore, perception of ability to make decisions about social justice and having a safe environment increased over the course of the training, from 58 to 81% and 69 to 81%, respectively; however, these increases were not significant based on chi^2^ tests (Supplementary Figures 16, 17; Table 3). These increases were most notable for girls compared to boys and lower grades compared to higher grades. After the training, 100% of non-people of color felt like they could make decisions about social justice issues, whereas the percentage for people of color stayed the same at 62% (Supplementary Figure 16). In a follow-up interview, a youth discussed their capacity for change and indicated that age may be a barrier to being more impactful and said, “Well, I think I’m a little too young, but I think just being on the youth advisory board and being here, I think that helps a little.”

Many participants stated that they are open to learning new things, changing their minds, and taking responsibility to create a better environment. A youth in a focus group said, “It means we’d better fix this place up, or we’re all going to die early.” In a focus group, one youth shared,

[…] *it’s just, it’s good to learn a lot about what’s going on, and it’s good to be cautious of what you are learning about and knowing about. And I just think it’s a good thing learning more about what you have not learned about.*

#### 3.1.4 Community change and collective action

A major focus of the training was translating environmental knowledge into action and building youth capacity for changing their communities. Activities that focused on EJ and the EMH interventions helped frame action and solutions at multiple scales. Most of the examples of community change and action that young people suggested were at the intrapersonal level, indicating an existing emphasis on individual action, consistent with the literature on environmental health and action (22, 45, 70, 71). Environmental science communication was also a form of action that was emphasized by youth.

In focus groups, 75% of participants (*N* = 40) mentioned a solution; 32% identified a solution at the cultural level, whereas 58% mentioned an intrapersonal solution (such as personal actions to educate oneself and using less water) and 52% a community level solution (Figure 2). A youth said,

*[…] I think like not so much just art, but a lot of people, graffiti in Globe and Miami, and maybe if they can change that graffiti from being bad, they can make it inspirational. And I think Globe and Miami are good towns. They have historical buildings. A lot of people would want to live here. It’s just it’s so dirty and full of substances that like if I was from say the Valley and I came down here, I would not want to live here.*

Solutions discussed in the follow-up interviews spanned all levels except public policy, with all 11 youth mentioning intrapersonal solutions (Supplementary Figure 18). EMH posters had a relatively even spread of solutions because the activity asked students to describe actions and interventions at each level of the model, except culture (Supplementary Figure 18). Several posters had intrapersonal decisions incorrectly categorized to interpersonal or community levels, perhaps indicating the limitation of strict definitions of interventions. In posters, solutions like the following came up:

*When there is a trash can throw it away.*

*DO not stop caring!*

*Have companies donate money to people who lost things in the fire (reparations)We can hold peaceful protest that are based around the smog companies produce and the companies dumping their trash wherever they want.*

*We can introduce more laws that prohibit companies from dumping their trash everywhere besides specific dumps along with having harsher punishments for companies who break said laws.*

*We could start a neighborhood [watch] to watch for strays dogs (watch dogs).*

These examples highlight that youth are thinking about environmental health in complex, systemic, and intersectional ways, consistent with existing literature on youth environmental organizing (22, 72, 73). In a focus group, a youth talked about their perspective on the current state of environmental policy and the importance of supporting marginalized communities.

*I think it’s a kind of middle ground for me. Some of these laws I’m very happy with, and some of them I’m very unhappy with. I feel personally that there should be more laws that are protecting things like groups of color and groups of low-income places. And there should be more laws telling us that we should get the information of what our land is, what’s going on. I feel like that should be something everyone gets. I think everybody should have a right to know what’s going on in the world.*

Photovoice submissions highlighted community-level solutions focused on infrastructure and urban development. One-fifth of Photovoice submissions coded to solutions were at the culture-level, indicating that something larger than one entity—government, law, or person—is necessary for change.

Youth Advisory Boards have served as a place-based continuation for environmental science and action after the weeklong summer trainings. They further EHL and community action through community building, science communication, and local advocacy. Of the 42 youth who earned micro-credentials (completed at least 75% of the summer training), 11 joined advisory boards. In January 2025, one summer training participant joined their community’s YAB after participating in a follow-up interview.

### 3.2 Critical consciousness

Throughout the training, youth were asked to reflect on themes of environmental and health justice, drawing connections to both systems of power and resistance/action. We analyzed youth data in terms of critical consciousness (43). In general, GM youth’s critical discussions focused on racism and other systems of oppression in detail. HW youth’s critical discussions focused on an oppressive community culture of silence and stubbornness. We analyzed quotes referring to power to understand the youth’s critical consciousness and the impact of the training. While youth generally discussed EJ as environmental issues in the community, some were able to comment in depth about systems of power and sociopolitical identities like age, race, class, and Indigeneity, and connect them to public health issues. Power and sociopolitical identity were often discussed in the context of environmental harms or obstacles to action. There were few instances of youth discussing power and identity as a source of pride or empowerment.

#### 3.2.1 Adults and youth power

Based on focus groups, follow-up interviews, participant observation, and conversations with youth participants, it became clear that a dominant culture of adult power and control shaped the limits of their imagination and capacity for enacting change. Participants made statements about how community members generally do not care, are unwilling to learn new things, and intentionally harm the environment, highlighting what other scholars have identified as colonial apathy and climate doomism (45). Youth participants cited these as obstacles to achieving EJ and solving important issues. Their points demonstrate critical reflection on age, power, and decision-making in their communities. In a follow-up interview, a participant remarked on youth power specifically and said, “Isn’t the point of an advisory to make better decisions as youth? I’m not sure how much control the youth advisory has over different stuff.*”* Youth detailed that some people in the community stubbornly stick to negative behaviors even when they know they are wrong and alluded to adults not wanting to leave their comfort zone. Youth highlighted shame, maintaining the status quo, and resistance to change as key factors in community members, celebrities, and government leaders shirking responsibility and accountability. One youth in a follow-up interview said,

*I feel like some people are a little too stubborn to see the problems. And stubborn people are probably the biggest problem because they do not want to admit that something’s wrong with it. And they do not want to admit that some things actually – that some things need in this town to be fixed.*

In a focus group, a youth said,

*Following up on what [they] said, I also think there’s a whole bunch of older generation people, like forties, fifties, around there, that just throw out their trash from their windows and throw it anywhere, especially if they are using substances, then it’s more likely that they’ll just do that, because they obviously do not care about anything like that. All they care about is their needs and all that.*

Some youth had an expansive understanding of someone’s motivations for action and inaction. They highlighted that people’s racist upbringing would be difficult to counteract, pointing to the influence of normalizing harmful ideas and behaviors such as racism and pollution, further highlighting the youth’s critical reflection. One youth cited one of the lessons on EJ and discussed the importance of how people were raised. They said,

*Like one of the videos in here said, some people might just be born with racist parents, and then that’s how they grew up. They think that’s like regular. And then obviously it’s not. So, then they do not know what’s wrong until they get old, which then by that point, they might just not care.*

Another youth said,

*I also feel like some people just like – they feel like they’d just rather not talk about it, because it can be an uncomfortable topic to them. Like they just do not want to accept that like it is a racial thing, or it could be like a low-income thing.*

In a follow-up interview, a youth reflected on the complexity of being a young person who may not be as educated or may be negatively influenced by social media, but still having good ideas that need to be heard, demonstrating critical reflection and political efficacy. Overall, this youth identified a culture that diminishes youth, their experiences, and knowledge. But there was a note of hope in their comment, that they believe in their community’s capacity to change, sharing,

*And one of the challenges of my community is, a lots of the people here are very stubborn, in the Globe Miami Claypool area. It’s really hard to sway them because it’s been so long since we have changed. And with the younger generation, a lot of people do not want to listen, because, you know, we are kids, we do not have much to say, we are uneducated, social media is rotting our brains, things like that. But it’s like, the more we talk about it and if we try to persuade certain people, it’s hard to – it’s almost like infecting them.*

*We have to get one of them to start talking about it to the other people, and then it finally is, like,* “Oh, maybe we should listen to them.” *So, I think outreach is one of the difficulties in my community. It’s so hard to get people to listen, but when they do, they catch on quick.*

#### 3.2.2 Government and institutional responsibility

Youth demonstrated critical reflection and political efficacy skills most when discussing the role of government and industry in EJ issues. They pointed out the culpability of leaders and corporations, describing how these groups are highly capitalist and value profit over human health, especially marginalized communities, a key component of global youth climate strikes (26, 72). One group explored burning buildings in their EMH poster, and wrote, “I don’t want to work for the government, I want the government to work for me,” (Figure 4). Further, in a focus group, a youth reflected,

*I think government plays a huge part in hearing other people’s opinions. If the government does not care about the people, then what kind of government is ruling us, right? So government plays a really big part. And the people also play a huge part. It’s like a conversation. It’s a two-way thing. It has to be a - there has to be an instigate and there has to be a response.*

During a focus group a youth stated,

*I think there’s definitely a big issue with industries, because there’s a lot of capitalism in America, and most people are just like, money, money, money, money. And environmental justice is something that’s kind of off to the side, you know? The government is focusing on bigger things, like homeless, and money, and helping communities. But environmental justice just kind of gets shooed away. And I think these companies do not really care about the environment, and they are more just like dumping it here is so much cheaper, and this helps us so much more. We do not care about the people living here.*

Another youth in a follow-up interview said,

*And our water – we just believe our – like our companies that take care of it, whether or not they say it’s good for us or not. But none of us can really have access to like checking it. We just naturally believe what they say.*

One youth cited colonial land theft from Indigenous peoples as a driver of environmental injustices and commented on the inequity of making Indigenous peoples buy back that land.

*Well, from my research that I have done we are the ones who took the land and who are polluting this land from the natives that had this land. And for them to have it back, we were making them pay […] money for it. In my opinion, I believe that we should at least split the land 50/50 and try to take care of it as best as we could together, instead of having this revolution or war of who owns the land and who does not.*

GM youth particularly elaborated on the downstream impacts of compounding injustices, indirectly connected to the environment. One youth highlighted that the San Carlos Apache Tribe does not have adequate lighting on the roads because they are under-resourced, posing a higher risk for car crashes. Another GM youth described the negative impact of the for-profit healthcare industry in the United States, highlighting how people in lower socioeconomic classes are less likely to see doctors because of the cost, a positive feedback loop resulting in worse health outcomes. A GM youth succinctly discussed the intersections between incarceration, colonialism, and pollution, remarking,

*I’ve also seen that, too, like with the reservation. I live down from the elementary school, so sometimes they have like prisoners go over there and clean up the streets and stuff. But if you go out to the reservation or even Miami, you can see they do not get as much attention as the other places. Like more of the low-income areas or something, they definitely do not get as much attention. They do not care about it as much.*

In a focus group, when discussing environmental issues and health issues like COVID-19, a youth described how government leaders can misuse science to harm communities.

*A lot of the other leaders, government, or stuff like that, are really ignorant when it comes to health issues, along with lead in the water, or air pollution, or anything really bad that could affect cities. And so when something does happen, they are like, oh, let me see the test, and they give you the evidence, and they are like, no, that cannot be right. Like our town is perfectly fine. And they shut it away until it actually kills half of the population, and they finally see how bad that is.*

#### 3.2.3 Culture of silence and punishment

Youth demonstrated critical reflection and critical action when discussing living in a culture of silence. During the training, youth consistently brought up intersectional understandings of environment, health, pollution, and justice, challenging adults to facilitate change at all levels of the EMH and name structural oppression as a root cause of local environmental issues. This was also discussed by a group of youth from northern British Columbia, Canada in a photovoice project about futures and environmental justice (73) and youth from climate justice organizations across the United States (27). Despite feeling like they are not allowed to talk about certain environmental or health issues due to social stigma, youth across the globe talk about them regardless of adult repression. In a focus group, a participant expanded on what another youth said, stating,

*Adding on to what he said about people not wanting to do it because of social stigmas, he’s exactly right. No one wants to come out and just talk about this stuff with a bunch of people, because they think that they’ll think they are stupid. But in a group like this where we have all been together for a week, it’s really easy to talk about it.*

Some youth were also grappling with what punishment and consequences should look like. Many of the statements considered regulation, community oversight, and increased fines. Some youth thought that the carceral system would support environmental clean-up and justice efforts, discussing jail time for corporate polluters or general punishment for individual anti-environmental behavior. On their poster about littering, one group said, “Cops should be at parks and other local places where more littering happens.” Others were more critical of punishment and consequences, discussing some of the broader reasons why people pollute. They identified more holistic ways to support people in making healthy decisions and taking environmentally conscious actions, rooted in ideas of transformative justice and a framework of abolitionist environmental justice (31). A youth said, “*Instead of making trash picking up, instead of making it a punishment, I feel like – if you teach it like younger on that it’s like a reward, like you are cleaning the earth, you are helping the environment.*”

In a conversation during the focus group about the intersection of drug and alcohol use with care for the environment, several youth described non-carceral alternatives to punishment. One youth stated,

*Based on what all of them said, I feel like they should get in trouble, but not as bad, because obviously, they are going on probation, and that goes on their record. And I feel like if they are self-medicating, if there’s a big problem that they have, like let us say there’s stuff going on at home that they are doing, and they are using that to self-medicate, then it would obviously just make more problems for them, and then it would go on their record, and they would not be able to get into good colleges. It would show on job applications. And then it’s just going to be hard down the road for them. So, I think it should be less strict […] I do not know how to put it, but I think they should add like groups to help them, and not put it on their record. But obviously, if it’s not their first offense, it probably should. If they do not seem to care about it, then it should. But like if you see that it’s like a problem, if there’s problems at home, they maybe should be put in a group instead.*

### 3.3 STEAM in action training reflections

**Figure 8:**
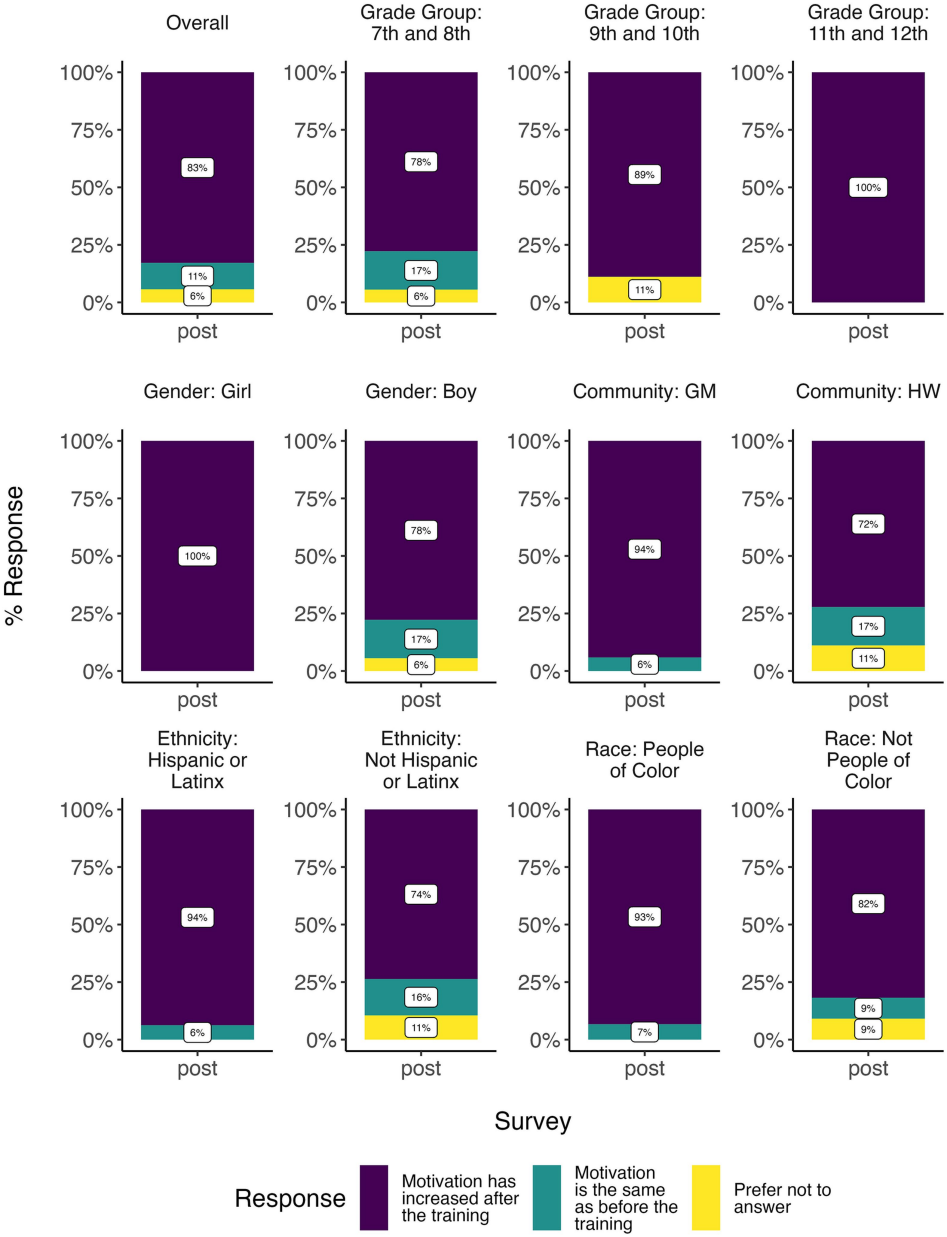
Bar plot summarizing post survey results for 36 youth who took both the pre- and post-surveys, for motivation to take action as a result of the training, demonstrating environmental health literacy—self-efficacy and community change.

When asked if their motivation to take action increased as a result of the training in the post-survey, 83% (*N* = 36) of youth overall said it did (Figure 8). A greater percentage of GM youth (94%), girls (100%), higher grades (89% for 9th and 10th, 100% for 11th and 12th), Hispanic or Latinx youth (94%), and youth of color (93%) said their motivation to take action increased because of the training (Figure 8). The mean satisfaction with the training on a scale of 1–10 was 8.74 (*n* = 34; Supplementary Figure 19). Girls (*p* = 0.068) and Hispanic or Latinx people (*p* = 0.020) were generally more satisfied with the training than boys and non-Hispanic or Latinx people (Supplementary Figure 19; Table 3).

Informal science education can effectively create learning environments, even for youth who are not initially interested in science. The summer trainings created a unique learning experience that the youth said they could not get at school (46, 74). One participant in a focus group said,

*I’ve never, like ever in my life been a fan of science, because I always thought it was just not my decision. I’m more of an art person. But I’ve always been a fan of the environment, the trees, the land, water, all that kind of stuff. So coming here and actually seeing all that’s involved within science and the environment was actually pretty cool to me. I learned a lot more about the environment than I would have ever done in school, because they do not really do that much anymore. So being here was definitely a treat […].*

Another student discussed seeing a future career in the environmental science field after initially joining the program because of monetary compensation. They said,

*[…] when I started and – the only reason I really joined was for the money. But when I was in there I was like,* “This is pretty cool.” *So I’m like – it was like,* “Yeah, I like this. This is something I could see a future in.” *Like environmental science.*

Many of the youth remarked about how helpful, understanding, and engaging the facilitators were. It was important that the educators were friendly and relatable, and made efforts to not replicate a traditional, hierarchical classroom structure. Building familiarity and trust between students and facilitators was pivotal in some students’ experience of the training and allowed youth to be more engaged, curious, and vulnerable (75). One youth in the focus group commented on the facilitators and said, “You acted like the cool aunts and uncles”. Another HW youth said,

*They were all so young and they knew some of the trendy social media stuff and they were just very helpful as well with learning. And if we did not get something they would come and help us and all that.*

A youth in their follow-up interview said,

*One of my favorite things about the training is that, I do not remember, I do not know how long it’s been since you have been in high school or things like that, but usually, there’s a very obvious divide between people, there’s certain groups and things. But it felt like even if, like, I was being annoying or loud or other people were being annoying and loud, we were all building a relationship. And you could see, as time went on and people got more comfortable, to present and put their personalities on their presentations. […] I really enjoyed building bonds and getting to talk to different people and hearing their ideas and the banter and trying to figure out what really is right or what we should do.*

Nonetheless, there were still aspects of the training that were complicated for students. The training relied on slide show lectures to deliver content, followed by group work or interactive activities, with mixed receptions. Based on facilitator observations of participants, youth were often bored by lecture presentations unless they were highly interactive. At times, it was challenging to keep the youth’s attention and properly share instructions, which is particularly important for hands-on environmental monitoring activities, where youth could be handling hazardous materials. Notably, the training did not exist in isolation from the rest of life. Friend groups, bullying, previous traumas, and family/community challenges were still present during the engagements. Relationship building between youth and facilitators allowed adults to deepen their understanding of students, supporting their education, engagement, and participation in the program.

## 4 Discussion

### 4.1 STEAM in action training

This training was created within the anti-racist FoK frameworks—a strengths-based way of honoring youth’s experiential/community knowledge (37–39). Activities and lessons were designed understanding that young people in rural EJ communities possess a high level of knowledge directly relevant to environmental health concerns; our data confirmed this. We aimed to create a space for young people to learn environmental health skills and to uncover and honor their experiential knowledge. This learning supports young people to translate their expertise into imagining and creating new futures for themselves and their communities. Limitations of this work include the small sample size (*N* = 49), overrepresentation of select partnering communities, and the place-specific context. However, the specific environmental and social context is what provided the impetus and crucial backdrop and foundation for the effort. Lastly, using a mixed-methods approach, we analyzed 9 evaluation tools (totaling over 200 individual artifacts) to create a thorough understanding of the outcomes associated with the “STEAM in Action” trainings.

It is important for educators to practice Wholistic Science Pedagogy, which is an approach where educators commit to self-awareness, science as transformative, restorative practices, and their students’ social emotional wellness (76–78). It was crucial that the program staff were relatable and shared social identities with the young people. Most of the program staff were people of color, most were women, several were queer/trans, some were from the youth’s communities, most had previous teaching or facilitation experience, and all strove to speak to young people as equals. These identities and values helped foster genuine relationships and create a comfortable environment where young people could learn. In part due to shared identities, staff were attuned to the emotional and physical needs of youth, not just the intellectual ones. To that end, throughout the summer trainings, we provided attendees with a variety of snacks and meals, fidget toys, affirmations altar, incorporated mindfulness and somatic activities, breaks for recreation like basketball and soccer, and games, daily. These unstructured moments reinforced relationships among youth and between staff and youth, creating a supportive environment that made it easier for youth to participate in the training. We also integrated guest speakers and field trips. To celebrate the end of the training, we hosted a showcase, graduation, and pizza party for the youth, their friends and family, and community members. Future trainings should focus on continuing to build family and community support for youth informal science education. Others who plan to host youth trainings should co-create trainings with community members and implement interdisciplinary approaches to holistically meet the needs of young people and practice a culture of care and reciprocity as embodied by the Charles Roundtree Bloom Project based in Yanaguana/San Antonio, Texas (31). This project contributes to an abolitionist environmental justice framework rooted in critical Black and Indigenous studies that calls for communities to undo settler-colonial carceral logics in part through healing relationships to people and land, leading to abolition and environmental justice (31, 79–81). Educators *must* be versed in critical and intersectional frameworks, adultism, and trauma-informed teaching; otherwise, they will reproduce the same issues of the formal education system, such as marginalization, censorship, adultism, white supremacy, and youth identity erasure, especially for students of color (31, 74, 82).

Several activities stood out as unique and successful ways of supporting youth EHL and critical consciousness. Our use of Photovoice allowed young people to share a glimpse of what mattered to them in their lives. To stay true to the intention of the method, the Photovoice submissions have also been shared with the larger community as a way to celebrate young people and center their needs and knowledge in local environmental discourse (58, 59). A project based in unceded Coast Salish territories in so-called British Columbia, Canada highlights the usefulness of Photovoice in empowering youth to share critical, rural, Indigenous, and place-based stories that are often left out of the environmental movement (73). Similarly, we use Photovoice to help elevate the issues critical to rural youth in environmental justice communities. During the youth co-authorship module in August 2025, the youth spent 2.5 h analyzing the selection of Photovoice submissions used in this paper, commenting on how interesting and compelling the photos and captions were compared to the other datasets. One youth reflected that looking at the Photovoice submissions humanized the data and created a personal connection to the content. This highlights the effectiveness of Photovoice as a tool for communicating environmental concerns and centering youth voice in the discourse. To see Photovoice data, please go to: https://explore.steamaction.arizona.edu/Photovoice.

The environmental monitoring activities, where the youth collected samples from their own homes and analyzed them using DIT methods, paired with data interpretation and visualization were also impactful. Research shows that hands-on environmental monitoring can increase participants’ understanding of pollution and their stake in public health (53, 83). Youth remarked on how they felt like scientists, wearing personal protective equipment, working with real samples, and documenting data. Youth presented their visualizations and conclusions to the training cohort during “Data Discos,” allowing them to practice communicating their results and celebrate their efforts as a group (Figure 5). The “Data Disco” was intentionally designed as a celebratory-style event where music played while youth walked up to share their results. This celebratory format transformed scientific communication into an engaging and affirming experience, allowing students to showcase their work with pride. It helped build confidence, reinforced the value of their contributions, and created a supportive, memorable atmosphere that emphasized joy and community alongside data analysis, contributing to continued youth engagement (71). The results of the pre- and post-surveys confirmed that self-efficacy for environmental monitoring increased as a result of the training (Figure 7; Table 3).

Our frameworks of EHL, FoK, and EJ were also critical to the program’s success because they demonstrated that environmental knowledge could be used for action and social change. An informal science education model was essential for us to explore EJ topics not often covered in formal educational spaces. When compared to formal education (like school) youth described the “STEAM in Action” training as more interesting and fun. However, we received feedback from some youth that aspects of the training, particularly slideshows and lectures, were boring and felt like school, potentially limiting engagement and learning. Future work should continue to operate in informal, welcoming settings to support critical consciousness for youth to learn about EJ topics in hands-on, culturally relevant ways that emphasize action in addition to learning.

One of the planned outcomes of the summer 2024 trainings was to set up YABs in our partner communities. At the first few meetings, the advisory board members established their mission statements, governance models, board names, ground rules, and goal membership numbers. All YAB activities are designed to support the project’s YAB aims around (1) members’ unique environmental health perspectives, (2) representing community perspectives, (3) fostering communication and critical thinking skills, (4) providing a “brave space” for environmental health discussions, increasing youth interest in environmental health sciences, and improving their scholastic trajectory, (5) becoming intrinsically and extrinsically motivated to share information and take action, and (6) providing a platform to address culturally nuanced power dynamics. Across communities, over 20 YAB meetings have been facilitated where youth have identified points of pride and areas of concern, researched locally identified issues, prepared infographics and presentations on these topics, engaged in personal reflection, co-designed STEAM in Action’s data explorer website, and contributed to an intergenerational data report-back celebration. Future work will analyze the experiences and outcomes of youth and adults involved in YAB activities.

### 4.2 Environmental health literacy and critical consciousness

A goal of the STEAM in Action program is to increase the EHL of rural youth in EJ communities to support community action. The mixed methods, informal science education approach to our training was pivotal in building youth EHL. Providing multiple pathways for learning allowed youth with different interests and learning styles to engage with the material. High-impact learning experiences such as environmental monitoring, arboretum field trips, Photovoice, and hands-on science projects diverge from traditional classroom environments that prioritize lectures and rote memorization over learning through lived experiences. Youth remarked throughout the training how much more interesting science was during the training compared traditional classroom. These efforts helped them in identifying issues/solutions and continuing their training with the research team via advisory boards and other engagements.

Allowing youth to express themselves in multiple artistic ways was essential to improving their EHL. Art allows people to engage embodied knowledge, learning through their body senses, which can support making sense of complex, political, and emotionally charged environmental phenomena (49, 84, 85). Artistic approaches to EHL have also been shown to support memory recall and action (49). In posters, presentations, data visualizations, and Photovoice, youth incorporated color, characters, memes, drawings, and movement to communicate their ideas. Their drawings and artistic renditions of data and environmental health topics reflect confidence and self-efficacy, while demonstrating increased EHL.

While youth demonstrated increases in awareness and knowledge of environmental health topics and self-efficacy for learning and doing science, the community change and collective action aspects of EHL were less prevalent, demonstrating less growth in critical consciousness than EHL. The differences in how youth discuss areas of concern and solutions reveal their spheres of influence, control, empowerment, and perhaps political analysis. Many youth responses highlighted pollution, culture, and individual actions as areas of concern. Interestingly, mining companies were not mentioned as often as expected, possibly as a result of the normalized, yet tense presence of mines in the communities. Young people appeared more inclined to highlight issues that they knew more about, more directly impacted them, or were less contentious, such as abandoned buildings and fires. Most youth discussed areas of concern related to a community and societal culture that is unsupportive of environmental health justice, highlighting societal stigmas, littering, and governmental failures. However, the solutions young people proposed did not always match the issues they identified. The disparity in responses suggests that while youth can identify problems, they may not know how to move forward as a collective to change society and the culture or have the power to enact the solutions they want (21, 22, 86, 87). Building capacity for collective action to affect community and systems change emerged as an area for growth in our trainings and is the mission of the ongoing YABs.

It can be difficult for youth living in EJ communities to acknowledge the unique sociopolitical dynamics at play in their communities; this training increased the youth’s critical consciousness and EHL and affirmed youth’s existing ideas by teaching EJ concepts and framing action and interventions using the EMH. A study of the Youth Leading Environmental Change project across six countries (Uganda, Bangladesh, India, Canada, Germany, and United States) similarly observed that their training increased environmental awareness and made existing youth understandings of environmental justice more concrete (44). It was also important to facilitate a welcoming environment for youth to ask questions about systems of oppression like racism and capitalism, which can be uncommon in rural company towns (88, 89). However, even though we were able to discuss certain sociopolitical issues, “STEAM in Action” was not immune to the impact of social oppression. We observed significant differences in EHL and self-efficacy by grade level, gender, and ethnicity, highlighting the importance of research with youth that considers the sociopolitical contexts that they live in by acknowledging histories of oppression and supporting marginalized peoples in culturally responsive learning (90, 91). Importantly, we observed that girls had lower self-efficacies for learning and doing science, but had similar increases in self-efficacy to boys, indicating that girls and boys came into our training with different confidence levels, but gained similar learning throughout the training (Supplementary Figure 12; Table 3). The underrepresentation and discrimination of girls in STEM fields, especially girls of color is widely studied (78, 90, 92, 93), a structural issue contributing to why girls in this study initially self-reported lower self-efficacies than boys. We did not observe major differences in EHL by gender. Other EJ efforts such as the HERMOSA project highlight the importance of safe and strengths-based youth spaces in increasing EHL and critical consciousness, particularly for youth of color (94). “STEAM in Action” joins many other youth-centered projects that emphasize the need for participatory research to be flexible with youth, approach leadership development and action with a critical lens, to address racism and sexism, and to be attentive to the youth’s various needs (17, 65, 90, 94–96).

Informal science education and community-university partnerships in “STEAM in Action” help proactively address the topics youth find relevant. Further attention should be paid to increasing youth’s ability to identify and discuss the systemic injustices impacting their communities, paired with growing capacity to think of and enact corresponding structural solutions. Part of these efforts may include directly supporting youth conceptions of culture and race, which can be complicated in multi-ethnic white, Latinx, and Indigenous communities (90, 97), especially for rural Arizona mining communities.

### 4.3 Resisting a culture of silence and punishment

Young people described living in a culture of silence, largely governed by corporate interests and adults’ resistance to change. It is controversial to discuss pollution from the ASARCO and Freeport McMoRan mines (examples brought up by students) and to critique the government. Youth perceive that teachers do not care about them, and results from our teacher surveys and listening sessions show that educators cannot easily teach environmental science or EJ curricula. The culture of silence means that young people are often forced to navigate pollution and resultant environmental health concerns without sufficient support. The impacts of adultism have been written about at length in other youth environmental activism efforts with young people calling attention to adults presuming youth incompetence, to co-optation of radical movements, to belittling youth concerns, to the need for sustained support structures, and to intergenerational collaboration (24, 25, 27). The dearth of youth power further robs young people of dreaming of their own futures. Their livelihoods and health are strongly impacted by governments and corporations, a classic trait of a company town, often rural communities, built around a particular form of resource extraction. Sicotte and White describe how in these communities, capitalist and racist corporations dominate the social, economic, and environmental landscapes of a town, thereby controlling what people do, say, and learn (88, 89). It is vital to have a diversity of economic resources in a community, so people are not constrained to narrow funding sources, like a single mining corporation. In addition to monopolizing economic opportunities as a form of control, companies also use the police, military, and violence to help keep their power (98–100). Rural mining towns in the US can be highly surveilled and policed places, often with disproportionate police presence and high rates of incarceration used to keep corporations in power (88, 101–103). In 2024, Arizona had an incarceration rate of 710 per 100,000 people, the 17th highest ranking state in the US (104). A strict company culture is not ideal for FoK or Community Cultural Wealth to perpetuate and grow, especially for young people seeking empowerment. Instead, we observed how a culture of silence and punishment can potentially limit EHL, critical consciousness, and community action, all of which pose a threat to company and government control.

Punishment and consequences were tensions that young people surfaced in various ways during the trainings. Youth opinions varied on how to treat polluters, especially depending on who was polluting. Some youth advocated for increased policing and imprisonment of people littering, with one youth describing how he wanted to potentially be a cop. While others, especially those personally impacted by the criminal justice system, opted for more transformative approaches to reducing harm. Youth described the importance of understanding all the reasons for someone’s behavior and working to support the individual in making different decisions instead of imprisoning and punishing them. Future work should take into account abolitionist environmental justice frameworks (31) and specifically create training activities for youth to unpack the relationships between carceral logics, abolition, and the environmental movement. However, when discussing corporations and governments, youth were supportive of increased fines and regulation. But youth also highlighted that they lack trust in the government or corporations to take their concerns and public health into consideration. According to anti-colonial and critical environmental justice scholars Liboiron and Pellow, if we continue relying on corporations and colonial governments for pollution limits, fines, and imprisonment, we may only reproduce the cultures and institutions that created pollution in the first place, particularly for rural EJ communities (2, 105, 106). During the youth co-authorship module, the youth emphasized the importance of having collaborative, diverse, and artistic interventions at multiple scales, particularly the culture and community levels. Other global youth-led movements such as the Palestinian Youth Movement and specific efforts for Palestinian youth to connect to the land also center the need for community organizing and collective action over passively relying on adult politicians, corporations, and colonial governments to make benevolent decisions in support of liberation movements (107–109). Future work should focus on cultivating critical conceptions of penalties/repercussions in rural communities and supporting young people in dreaming up transformative solutions to environmental harms that do not recreate the conditions that created them.

At the core of much youth EJ and EHL work is the power imbalance between adult decision makers and young people with unmet needs and ideas for change in their communities, i.e., adultism (22, 25). If EJ and EHL efforts do not work to dismantle the structures that disempower youth, increasing youth literacy will not result in social change. The Community Cultural Wealth model provides a strengths-based, intergenerational, multiscale framework to support the empowerment of rural marginalized communities. It highlights that even though marginalized communities may feel powerless in the face of systemic oppression, they have substantial cultural capital that can be honored and cultivated to change systems (41). Rooted in the values of transformative justice, interconnectedness with the environment, reciprocity, and youth power, intergenerational EJ projects can restore cultures and hold corporations and governments accountable to protecting socioecological health (25, 110–112).

### 4.4 Hopes and recommendations

Young people part of “STEAM in Action” are pushing back against corporate control, a culture of silence, and adultism by learning and doing environmental science, speaking out, staying curious, and participating in intergenerational EHL activities. This project, particularly the Photovoice activity, allowed youth to shed light on taboo and inadequately discussed issues in their town. While young people felt some apathy and powerlessness when it came to changing their community for the better, highlighting the difficulty of changing systems and cultures run by adults, there was also a theme of hope through education and community advocacy. Nearly all the youth felt their motivation to take action increase as a result of the STEAM in Action training (Figure 8). This program provides a safe space for youth to engage in environmental health and EJ topics typically ignored, sidelined, or silenced in our partner communities. Young people need a safe and playful space to discuss EJ issues with a focus on action and empowerment that is centered on collective, not individual action and honors the emotional and political realities of environmental injustice (45). Adults and educators must create supportive and caring environments for youth to engage in EJ topics and to become changemakers in their communities.

A recommendation for replication of this work in other rural or environmental justice contexts include program co-creation with community members, which allows one to align content and action to community needs and sociopolitical contexts. We also recommend informal science education combined with intergenerational data report back, communication, and advocacy efforts. A mixed-methods, qualitative and quantitative evaluation approach can help one understand the complexities of learning and action, while honoring community narratives and lived experiences. Finally, we recommend building strong relationships with youth, honoring their whole person, and building youth community organizing skills to counteract the dehumanization and repression they experience. Future work should continue to study youth power and environmental health literacy, but expand to exploring regenerative and extractive economies, decolonization, abolition, and radical futurisms.

This study advances the field beyond existing community-based health literacy programs by identifying (1) the high level of literacy already embedded in the community, (2) the challenge of adultism in achieving environmental justice, (3) the importance of considering critical consciousness in literacy, and (4) the necessity of youth informing how environmental health data should be visualized to elicit knowledge building and change. The program also allowed youth to bring up controversial areas of concern and solutions in their communities, name their values, generate quantitative environmental monitoring datasets, and contribute to scholarly work through our hands-on youth co-authorship module, which are outcomes not limited to public health.

## Data Availability

The raw data supporting the conclusions of this article will be made available by the authors, without undue reservation.

## Footnotes

1
https://www.steamaction.arizona.edu/

2
https://www.steamaction.arizona.edu/trainings/youth

3
https://www.steamaction.arizona.edu/trainings/adults
