# Supplementary material for: Building youth power and environmental health literacy with environmental justice communities in rural Arizona
Source: Front Public Health. 2026 May 12;14:1733720. doi: 10.3389/fpubh.2026.1733720 (PMC13201490; doi:10.3389/fpubh.2026.1733720)
Supplement: Supplementary file 1 [file Data_Sheet_1.pdf]

STEAM in Action: Youth Training Survey

Thank you for participating in the STEAM in Action Training! We are interested in learning about you and excited to hear your ideas! In order to best serve you and your community, and to gather ideas to make this training even better, we ask that you complete this survey before AND after the training.

To receive your micro credential from the University of Arizona, you must complete this survey before AND after the training

Please provide your first and last name. ______________________

Part One: Environmental Justice, Public Health Issues, and Environmental Monitoring

Have you heard the term "environmental justice" before?

- ◯ Yes
- ◯ No
- ◯ I don't know

Think about when you've heard the term "environmental justice" before. Which of the following do you think best defines an environmental justice community?

- ◯ A community against equitable environmental protection
- ◯ A community seeking equitable environmental protection
- ◯ A community seeking participation in capitalism
- ◯ A community against participation in capitalism
- ◯ I don't know

Have you heard of a "public health issue" before?

- ◯ Yes
- ◯ No
- ◯ I don't know

Think about when you've heard of a "public health issue" before. Which of the following do you think best defines a public health issue?

- ◯ Issue related to preventing disease in individuals
- ◯ Issue related to technology advancement
- ◯ Issue related to career advancement
- ◯ Issue related to the overall health of individuals and communities
- ◯ I don't know

Have you heard of "environmental monitoring" before?

- ◯ Yes
- ◯ No
- ◯ I don't know

Think about when you have heard about "environmental monitoring" before. Which of the following do you think best defines environmental monitoring?

- ◯ Information that only companies can provide on how their business affects the environment
- ◯ Information that only individuals can collect to determine the risks in their environment
- ◯ Anyone collecting information to determine the impact of activities on the environment
- ◯ Anyone making claims (with or without evidence) about the health of the environment
- ◯ I don't know

Are you familiar with any environmental laws or regulations related to what people or businesses are allowed to do with or on land, water or other things in the environment?	◯ I am not familiar with any of these laws or regulations<br>◯ I am a little familiar with these laws or regulations<br>◯ I am aware that these laws or regulation exist, but I don't know what they are<br>◯ I am both aware and familiar with these laws or regulations<br>◯ I don't know
Please tell us more about any laws or regulations you know about and what in the environment they protect. Don't worry about using any official or technical words, feel free to use your own words to describe the laws or regulations you know about.	__________________________________

Part Two: Describe Your Community

Do you think that there are environmental problems in your community?	◯ Yes<br>◯ No<br>◯ I don't know
Please explain the environmental problems in your community that you know about. Don't worry about using official or technical words, use your own words to tell us about what you know.	__________________________________
Do you think that there are justice or injustice problems in your community?	◯ Yes<br>◯ No<br>◯ I don't know
Please explain the justice or injustice problems in your community that you know about. Don't worry about using official or technical words, use your own words to tell us about what you know.	__________________________________
Do you think your community has enough clean water for everyone to stay healthy?	◯ Yes<br>◯ No<br>◯ I don't know
Please tell us what you know about your community not having enough water for everyone to be healthy. Don't worry about using official or technical words, use your own words to tell us about what you know.	__________________________________
Do you think your community has enough clean air for everyone to be healthy?	◯ Yes<br>◯ No<br>◯ I don't know
Please tell us what you know about your community not having enough clean air for everyone to be healthy. Don't worry about using official or technical words, use your own words to tell us about what you know.	__________________________________
Do you think your community has enough healthy food for everyone to eat?	◯ Yes<br>◯ No<br>◯ I don't know

Please tell us what you know about your community not having enough healthy food for everyone to eat. Don't worry about using official or technical words, use your own words to tell us about what you know.	__________________________________
Do you think your community has enough healthy activities for everyone to do and have fun?	◯ Yes<br>◯ No<br>◯ I don't know
Please tell us what you know about your community not having enough healthy activities for everyone to do and have fun. Don't worry about using official or technical words, use your own words to tell us about what you know.	__________________________________
Do you think that you and others in your community are able to help make decisions about having a safe environment to live in?	◯ Yes<br>◯ No<br>◯ I don't know
Please tell us what you know about you and other people in your community not being able to help make decisions about the environment that you/they live in. Don't worry about using official or technical words, use your own words to tell us about what you know.	__________________________________
Do you think that you and others in your community are able to help make decisions about social justice issues (i.e., issues that are about the fair and equal treatment of everyone)?	◯ Yes<br>◯ No<br>◯ I don't know
Please tell us what you know about you and other people in your community not being able to help make decisions about social justice issues (i.e., fair and equal treatment for everyone). Don't worry about using official or technical words, use your own words to tell us about what you know.	__________________________________
Do you think there are environmental problems in your community?	◯ Yes<br>◯ No<br>◯ I don't know
Please tell us what you know about any environmental problems in your community. Don't worry about using official or technical words, use your own words to tell us about what you know.	__________________________________
Do you think that people in your community have different opinions or disagree about the environmental problem(s) that you wrote about in the last question?	◯ Yes<br>◯ No<br>◯ I don't know
Please tell us what you know about people in your community having different opinions or disagreeing about one or more environmental problems. Don't worry about using official or technical words, use your own words to tell us about what you know.	__________________________________

Part Three: Use the scenario described here to answer the following questions.

Imagine that your family is looking for a new home. You see an ad about a family-friendly housing development that will be built surrounding a park and a playground on land that is currently vacant. You think it looks nice and that the park would be great for siblings to play and for you to hang out with your friends. The ad says that the company building the homes will be at a community meeting, that you attend while other members of your family are unavailable. At the meeting, someone asks the person from the company if they are sure that the land is safe because a gas station used to be there. The person from the company claims that it is safe because the lab team for the company has visited the site and determined it to be safe for housing and a park.

Do you believe the claim that the person from the company makes about the land being safe?

◯ Yes
◯ No
◯ I don't know

Please tell us why you do or do not believe the person from the company. Don't worry about using official or technical words, use your own words to tell us about your answer.

____

What information or evidence would you want or need to know to help your family make a decision about whether or not you should move to the new homes?

____

If you worked for the company's lab team and were in charge of checking whether or not the land was safe, what actions would you take?

____

Part Four: Please complete the following ratings.

How would you rate your understanding of what is done in environmental monitoring (testing samples from the environment to see if it is safe)?

◯ 1 (no understanding)
◯ 2
◯ 3
◯ 4
◯ 5
◯ 6
◯ 7
◯ 8
◯ 9
◯ 10 (expert understanding)
◯ Prefer not to answer

How would you rate your ability to use tools and directions to test soil?	◯ 1 (I don't know anything about the tools or directions needed to test soil)<br>◯ 2<br>◯ 3<br>◯ 4<br>◯ 5<br>◯ 6<br>◯ 7<br>◯ 8<br>◯ 9<br>◯ 10 (I am an expert at using tools and directions to test soil)<br>◯ Prefer not to answer
How would you rate your ability to use tools and directions to test the air?	◯ 1 (I don't know anything about the tools or directions needed to test the air)<br>◯ 2<br>◯ 3<br>◯ 4<br>◯ 5<br>◯ 6<br>◯ 7<br>◯ 8<br>◯ 9<br>◯ 10 (I am an expert at using the tools and directions needed to test the air)<br>◯ Prefer not to answer
How would you rate your ability to use tools and directions to test dust?	◯ 1 (I don't know anything about the tools and directions needed to test dust)<br>◯ 2<br>◯ 3<br>◯ 4<br>◯ 5<br>◯ 6<br>◯ 7<br>◯ 8<br>◯ 9<br>◯ 10 (I am an expert at using the tools and directions needed to test dust)<br>◯ Prefer not to answer
How confident are you in your ability to LEARN science about reducing your exposure to dangerous stuff in the environment?	◯ 1 (no confidence)<br>◯ 2<br>◯ 3<br>◯ 4<br>◯ 5<br>◯ 6<br>◯ 7<br>◯ 8<br>◯ 9<br>◯ 10 (very confident)<br>◯ Prefer not to answer

How confident in are you in your ability to DO science about reducing your exposure to dangerous stuff in the environment?	◯ 1 (no confidence)<br>◯ 2<br>◯ 3<br>◯ 4<br>◯ 5<br>◯ 6<br>◯ 7<br>◯ 8<br>◯ 9<br>◯ 10 (very confident)<br>◯ Prefer not to answer

Part Five: Pre or Post Survey

Are you completing this survey before or after the STEAM in Action training?	◯ Before (or at the beginning of the training)<br>◯ After (or at the end of the training)
How satisfied are you with the STEAM in Action training?	◯ 1 (not at all satisfied)<br>◯ 2<br>◯ 3<br>◯ 4<br>◯ 5<br>◯ 6<br>◯ 7<br>◯ 8<br>◯ 9<br>◯ 10 (very satisfied)<br>◯ Prefer not to answer
How motivated are you to take action related to anything you learned about in this training?	◯ Motivation is the same as before the training<br>◯ Motivation has decreased after the training<br>◯ Motivation has increased after the training<br>◯ Prefer not to answer
What actions are you motivated to take?	__________________________________

Demographic Information
We are collecting demographic information from all of our participants to help us determine if we have representation of your community. Please note all questions are optional, and your decision to answer or skip a question will not affect you in any way.

Were you born in the United States?

◯ Yes
◯ No
◯ Prefer not to answer

Have you lived in your community for 5 years or more?

◯ Yes
◯ No
◯ Prefer not to answer

Are you part of the following Community Based Organizations? (Select all that apply)

☐ Environmental groups
☐ Youth group
☐ Museum volunteer/staff
☐ Library group
☐ Community food bank volunteer/staff
☐ Faith-based groups
☐ Health education centers volunteer/staff
☐ Community sports club(s)
☐ Other (Please specify)
☐ None
☐ Prefer Not to Answer

Please specify: __________________________________

Which of the following best describes you? (Select all that apply)

☐ Woman
☐ Man
☐ Agender
☐ Gender Non-Conforming
☐ Non-Binary
☐ Transgender
☐ Self-Identity (optional textbox)
☐ Prefer not to answer

Please specify: __________________________________

Which of the following best describes you? (Select all that apply)

☐ American Indian or Alaskan-Native (Please specify name of enrolled principal tribe(s). For example, Navajo Nation, Tohono O'odham Nation, San Carlos Apache Tribe, etc.)
☐ Asian American, Asian or Pacific Islander (Please specify. For example, Asian Indian, Chinese, Filipino, etc.)
☐ Black or African American (Please specify. For example, African American, Jamaican, Haitian, etc.)
☐ Hispanic or Latinx (Please specify. For example, Mexican, Cuban, Puerto Rican, etc.)
☐ Middle Eastern or North African (Please specify. For example, Arab, Egyptian, Palestinian, etc.)
☐ White or European (Please specify. For example, English, German, Irish, etc.)
☐ Other (Please specify)
☐ Prefer not to answer

Please specify: __________________________________

What is your age? If you prefer not to answer, respond N/A __________________________________

Please indicate your primary language.

◯ English
◯ Spanish
◯ Other (Please specify)
◯ Prefer not to answer

Please specify: __________________________________

Please indicate your second language.

◯ English
◯ Spanish
◯ Other (Please specify)
◯ I do not speak another language
◯ Prefer not to answer

Please specify: __________________________________

What is your home zip code? If you do not wish to respond, please write N/A __________________________________

What grade will you be in next school year? (Select one)

◯ 7
◯ 8
◯ 9
◯ 10
◯ 11
◯ 12
◯ Prefer not to answer

How many people live in your household?

◯ 1 ◯ 2 ◯ 3 ◯ 4
◯ 5 ◯ 6 ◯ 7 ◯ 8
◯ 9 ◯ 10 or more ◯ Prefer not to answer

Thank you for completing this survey, we hope you find the STEAM in Action training to be engaging and informational! Please include any additional information you want us to know about you or your community here, and then click "submit". __________________________________

Thank you and congratulations on completing the STEAM in Action Training! Please use this area to provide us with additional feedback about the training, or any other information you would like us to know about you or your community, and then click "submit". __________________________________

Install Date __________________________________

Participant Code __________________________________
