# Supplementary material for: Building youth power and environmental health literacy with environmental justice communities in rural Arizona
Source: Front Public Health. 2026 May 12;14:1733720. doi: 10.3389/fpubh.2026.1733720 (PMC13201490; doi:10.3389/fpubh.2026.1733720)
Supplement: Supplementary file 2 [file Data_Sheet_2.docx]

Supplemental Material 2 for Building youth power and environmental health literacy with environmental justice communities in rural Arizona

Kunal Palawat^1^, William Borkan^1^, Sanlyn Buxner^2^, Isabella M. Castañeda^3^, Sallie Choi^3^, Ted Choi^3^, God’sgift N. Chukwuonye^1^, Melissa Jaquez^1^, Miriam Jones^1^, Anastasia Mariscal^3^, Miracle Martinez^1,4^, Spencer T. McBride^3^, Carol Newbauer^1^, Caleb Ochoa^3^, Benjamin Quesada^3^, Maricela Quesada^3^, Raquel N. Quesada^3^, Iliana A. Samorano^1^, Felix L. Vincent^3^, Abigail Zettlemoyer^1^, Mónica D. Ramírez-Andreotta^1,5*^

Affiliations

^1^Department of Environmental Science, College of Agriculture, Life, and Environmental Sciences, University of Arizona, Tucson, AZ, USA

^2^College of Education, University of Arizona, Tucson, AZ, USA

^3^Youth Advisory Board, “STEAM in Action”, Arizona, USA

^4^Regenerating Sonora, Inc., Superior, AZ, USA

^5^Mel and Enid Zuckerman College of Public Health, University of Arizona, Tucson, AZ, USA

All authors except for first and last are listed alphabetically.

*Corresponding author: Dr. Mónica D. Ramírez-Andreotta; [mdramire@arizona.edu](mailto:mdramire@arizona.edu)
1177 E 4^th^ St, Shantz 429, Tucson, AZ 85719, USA.

| **5E Lesson Plan – Introduction to Environmental Science, Health & Policy** |
| --- |
| **Lesson objectives:**   - Analyze the relationships between humans, the environment, and earth’s resources - Explain the importance of equitable environmental health protections for all communities - Research a local environmental health issue - Collaborate and create a poster to inform others about a local environmental health issue - Communicate to others the source, effects, solutions, and policies related to a local environmental health issue |
| **Resources needed to teach this lesson:**   - A method for displaying and filling out a KWL chart - Technology to show the video clip linked below - Poster paper and art supplies - Laptops/computers for researching a chosen local issue |
| **Engage**   - Use a white board, poster paper, or a projected slide to begin a KWL chart   - Ask: What do you already Know about environmental health? (record on chart)   - Ask: What do you Want to know about environmental health? (record on chart) - Watch this video clip from the American Public Health Association (4:31): <https://www.youtube.com/watch?v=d0t1gW99fqg>   - Ask: What can we add to the K and W columns of the chart? (record on chart)   - Ask: What did you learn from the video that we can add to the Learned column? (record on chart) |
| **Explore**   - Discuss   - Ask: What kinds of environmental issues are you aware of in your community?   - Ask: Which issue(s) is/are most important to you and why?   - Ask: What are some ideas for solutions or remedies to the issues discussed? |
| **Explain**   - In a format that is convenient and comfortable (lecture, slides, discussion, etc.) convey the following four concepts:   - Studying the environment helps people to understand issues that might affect their health   - Governments use information gained from studying the environment to make laws and policies to protect people’s health   - Unfortunately, not all communities experience the same (or equitable) levels of environmental health protections; environmental justice seeks protection for all   - When citizens are informed and engaged, they can hold the government and other parties accountable for enacting equal (or equitable) protections for all communities |
| **Elaborate/Apply learning**   - Posters for Peers   - Directions for participants:     - Work in a small group, with a partner, or individually to identify an environmental issue in your community that you think your peers should know about     - Create an informational poster that could be hung up in your school to educate your peers about the issue you chose     - Include (use the internet to find information if needed; remember to use reputable sources!):       - The source of the issue       - The affects on human health       - Whether or not it is an environmental justice issue       - Possible solutions or remedies       - Current or needed laws/policies that would protect human health     - Include graphics and pictures to help your peers understand the problem and what actions they can take   - Provide poster paper, art supplies, a computer for basic research, and a collaborative environment where creativity is encouraged |
| **Evaluate:**   - Participants demonstrate they have reached the lesson objectives by sharing and describing their posters to the group |
