# Supplementary material for: Building youth power and environmental health literacy with environmental justice communities in rural Arizona
Source: Front Public Health. 2026 May 12;14:1733720. doi: 10.3389/fpubh.2026.1733720 (PMC13201490; doi:10.3389/fpubh.2026.1733720)
Supplement: Supplementary file 3 [file Data_Sheet_3.docx]

Supplemental Material for

Supplemental Material 3 for Building youth power and environmental health literacy with environmental justice communities in rural Arizona

Kunal Palawat^1^, William Borkan^1^, Sanlyn Buxner^2^, Isabella M. Castañeda^3^, Sallie Choi^3^, Ted Choi^3^, God’sgift N. Chukwuonye^1^, Melissa Jaquez^1^, Miriam Jones^1^, Anastasia Mariscal^3^, Miracle Martinez^1,4^, Spencer T. McBride^3^, Carol Newbauer^1^, Caleb Ochoa^3^, Benjamin Quesada^3^, Maricela Quesada^3^, Raquel N. Quesada^3^, Iliana A. Samorano^1^, Felix L. Vincent^3^, Abigail Zettlemoyer^1^, Mónica D. Ramírez-Andreotta^1,5*^

Affiliations

^1^Department of Environmental Science, College of Agriculture, Life, and Environmental Sciences, University of Arizona, Tucson, AZ, USA

^2^College of Education, University of Arizona, Tucson, AZ, USA

^3^Youth Advisory Board, “STEAM in Action”, Arizona, USA

^4^Regenerating Sonora, Inc., Superior, AZ, USA

^5^Mel and Enid Zuckerman College of Public Health, University of Arizona, Tucson, AZ, USA

All authors except for first and last are listed alphabetically.

*Corresponding author: Dr. Mónica D. Ramírez-Andreotta; [mdramire@arizona.edu](mailto:mdramire@arizona.edu)
1177 E 4^th^ St, Shantz 429, Tucson, AZ 85719, USA.

**Ecological Health Model Worksheet**

**STEAM in Action, 2024**

**Definition**

The ecological model (figure below) describes five levels of influence that determine health-related behaviors; each level is a potential target for health promotion intervention.^[[1]](#endnote-1)^ Ecological health models can be used to develop a comprehensive intervention approach that systematically targets mechanisms of change at each level of influence. They also can provide comprehensive frameworks for understanding the multiple, interacting determinants of health, or in this case, environmental health challenges.

**Activity**

Working in groups:

- Identify a local issue.
- Propose and describe an activity/intervention that can be completed at each of the five levels of influence to address the identified issue.
  1. Intrapersonal

- 1. Interpersonal
  2. Institutional
  3. Community
  4. Public Policy

1. Schneider, Mary-Jane. *Introduction to Public Health*, Jones & Bartlett Learning, LLC, 2016.*ProQuest Ebook Central*, http://ebookcentral.proquest.com/lib/uaz/detail.action?docID=4441707.
   Created from uaz on 2024-03-20 22:34:24. [↑](#endnote-ref-1)
