# Supplementary material for: Building youth power and environmental health literacy with environmental justice communities in rural Arizona
Source: Front Public Health. 2026 May 12;14:1733720. doi: 10.3389/fpubh.2026.1733720 (PMC13201490; doi:10.3389/fpubh.2026.1733720)
Supplement: Supplementary file 4 [file Data_Sheet_4.docx]

Supplemental Material 4 for Building youth power and environmental health literacy with environmental justice communities in rural Arizona

Kunal Palawat^1^, William Borkan^1^, Sanlyn Buxner^2^, Isabella M. Castañeda^3^, Sallie Choi^3^, Ted Choi^3^, God’sgift N. Chukwuonye^1^, Melissa Jaquez^1^, Miriam Jones^1^, Anastasia Mariscal^3^, Miracle Martinez^1,4^, Spencer T. McBride^3^, Carol Newbauer^1^, Caleb Ochoa^3^, Benjamin Quesada^3^, Maricela Quesada^3^, Raquel N. Quesada^3^, Iliana A. Samorano^1^, Felix L. Vincent^3^, Abigail Zettlemoyer^1^, Mónica D. Ramírez-Andreotta^1,5*^

Affiliations

^1^Department of Environmental Science, College of Agriculture, Life, and Environmental Sciences, University of Arizona, Tucson, AZ, USA

^2^College of Education, University of Arizona, Tucson, AZ, USA

^3^Youth Advisory Board, “STEAM in Action”, Arizona, USA

^4^Regenerating Sonora, Inc., Superior, AZ, USA

^5^Mel and Enid Zuckerman College of Public Health, University of Arizona, Tucson, AZ, USA

All authors except for first and last are listed alphabetically.

*Corresponding author: Dr. Mónica D. Ramírez-Andreotta; [mdramire@arizona.edu](mailto:mdramire@arizona.edu)
1177 E 4^th^ St, Shantz 429, Tucson, AZ 85719, USA.

**STEAM in Action, Summer 2024 (first year offering youth trainings)**

**Focus Group Script**

**Set up**

- Recording devices (2)
- Confirm all in focus groups have a signed assent and consent form
- Make sure chairs and table are informally set up, horseshoe, circle.
- Ask your facilitator partner to do participant observation, *taking notes without taking notes!*

**Thank you!**

- Thank you for your willingness to participate! Your responses will help us design environmental health education programs.

**Opening the chat!**

- Introduce yourself and the purpose of the conversation.
- Ramírez - I am an environmental health scientist and associate professor of Environmental Science at the University of AZ...

**[They will at this point already know us, so this can be brief!]**

- We’re so glad you are all here! It has been a wonderful week! We are now at the end of our training and it’s time for our focus group!
- A focus group is a research method where we discuss topics and answer questions in a group setting. Explain that they can share their opinions and that you will guide the discussion by asking the group to reflect on specific questions.
- This is a form of human research; remember those forms you and your parents had to sign!! **[Confirm all in focus groups have a signed assent and consent form]**
- Tell them what time the session will conclude.

**Safe Space**

Explain the ground rules below for the focus group discussion. These will set the tone and expectations for behavior so that everyone will feel safe and willing to participate.

- Participation in the focus group is voluntary.
- It’s all right not answer and questions if you are not comfortable.
- All responses are okay — there are no right or wrong answers.
- Please respect the opinions of others even if you don’t agree.
- Try to stay on topic; we may need to interrupt so that we can cover all the material.
- Speak as openly as you feel comfortable.
- Help protect others’ privacy by not discussing details outside the group.
- Allow time for questions, and then ask participants to introduce themselves.

**Getting started!**

- Please introduce yourself by your first name and/or kit #.
- We will be recording this conversation, please know these recordings will only be used by our UA research team to better understand how our training went and what we learned together.

**General prompts that can be used throughout:**

- What do others think?
- When someone agrees with another, ask them to elaborate “Why do you agree? Disagree?”
- When something interesting/compelling is stated, ask them to elaborate on why, what makes you say that? Was there something you saw or heard that you made you say that?

**If they are silent:**

- Ask for a pair-share and then share with group
- You can write on your white board and then raise up

**Random notes!**

- Can’t hear them – state: I really want to hear what you have to say, physically move to them, be silly!
- Be prepared to restate the question to provide clarity! Use an example/analogy that is different than our training.

**DO NOT**

- Fill in their words, don’t speak for them!
- Focus solely on one person.

**Questions (numbers in front of questions align with grant aim-hypothesis)**

1. 2-2.1: What do you know now about EJ that you didn’t know before? Any surprises? What does EJ mean to you?
   1. Prompts: Remember what we did on Tuesday and Wednesday? The video about the protest? With the data visualizations? Let’s just talk about what you learned!!
2. 2-2.2: Why do you think it is so hard to address EH and EJ issues?
   1. [Prompts: I know we talked about EH, but what are other social issues that are hard to solve? Why?
3. 2-2.4 and 2-2.7: Tell me about how or why social issues impact public and environmental health.
   1. Prompts: [Maybe use “SES” or another way to explain that since students might be unfamiliar with that term; also include any marginalized community]
4. 2-2.5: Did you learn anything new about the way the environment works and how it connects to health? If so, please tell me more about that.
   1. Prompts: Why did we collect soil, water and dust samples? Set up the air monitor?
5. 2-2.3: Based on how you all described how the environment works and how it connects to health, do you have any local or other concerns?
   1. If time, do you see anything uplifting or inspiring happening around you related to environmental health?
6. 2-2.6: What do you think about the current environmental laws about how land is used (resource extraction and redevelopment)? Do you feel protected?
   1. Prompts: Imagine a time when you did not have to wear a seatbelt! Seat belt laws protect us; can you think of an environmental law that protects you like the seat belt?
   2. What kind of laws would make you feel protected?

**[if time allows] At 5:35PM, must start ecological model!**

1. 2-2.8: Work through an issue together as part of the focus group. Ask group to agree upon the issue discussed and then discuss each area of the EH model: public policy, community, institutional, interpersonal, intrapersonal. **[~25 min needed! Handout the “Ecological Health Model_worksheet”]**
   1. ID a topic – based on their conversations and topic discussed throughout week, we will select the topic!
   2. Individual work on personal white board (artifact collection!)
   3. Create the combined ecological model of health

**Optional Questions!**

1. Has there been a shift for you in the way you view science and scientists, specifically EH? Tell me more about that
2. Are you feeling more interested in taking more science or government classes than you were before this training? What about joining a debate team or other extracurriculars related to EH or EJ?
3. Has your interest in STEAM careers changed since taking this training? In what ways?
   Prompts: [Are you thinking differently about what you want to study or do after high school?]

**Wrap up!**

- Last 2 minutes - Close the session.
- End the discussion by summarizing the main points.
- If there is time, invite participants to reflect on the main ideas, and ask if they have any additional thoughts to share.
- Thank the group for participating; let them know how the discussion results will be used. Explain program goals: “Our goal is to design STEAM education programs that engages 7th-12th graders and provides them with the tools, skills and support to document, reflect upon, and communicate issues of concern (through advisory boards and Photovoice); collect environmental samples (through co-created citizen/community science); and make sense of data so that their results can inform actions at the local, regional, and even national level (through data visualization and environmental communication)”
- Collect and save all notes and recordings, e.g., the Ecological model of health hard copy and/or photo of individual and group whiteboard.
- Ask if they want to receive the learning research results/follow up!
