# Supplementary material for: Building youth power and environmental health literacy with environmental justice communities in rural Arizona
Source: Front Public Health. 2026 May 12;14:1733720. doi: 10.3389/fpubh.2026.1733720 (PMC13201490; doi:10.3389/fpubh.2026.1733720)
Supplement: Supplementary file 5 [file Data_Sheet_5.docx]

Supplemental Material 5 for Building youth power and environmental health literacy with environmental justice communities in rural Arizona

Kunal Palawat^1^, William Borkan^1^, Sanlyn Buxner^2^, Isabella M. Castañeda^3^, Sallie Choi^3^, Ted Choi^3^, God’sgift N. Chukwuonye^1^, Melissa Jaquez^1^, Miriam Jones^1^, Anastasia Mariscal^3^, Miracle Martinez^1,4^, Spencer T. McBride^3^, Carol Newbauer^1^, Caleb Ochoa^3^, Benjamin Quesada^3^, Maricela Quesada^3^, Raquel N. Quesada^3^, Iliana A. Samorano^1^, Felix L. Vincent^3^, Abigail Zettlemoyer^1^, Mónica D. Ramírez-Andreotta^1,5*^

Affiliations

^1^Department of Environmental Science, College of Agriculture, Life, and Environmental Sciences, University of Arizona, Tucson, AZ, USA

^2^College of Education, University of Arizona, Tucson, AZ, USA

^3^Youth Advisory Board, “STEAM in Action”, Arizona, USA

^4^Regenerating Sonora, Inc., Superior, AZ, USA

^5^Mel and Enid Zuckerman College of Public Health, University of Arizona, Tucson, AZ, USA

All authors except for first and last are listed alphabetically.

*Corresponding author: Dr. Mónica D. Ramírez-Andreotta; [mdramire@arizona.edu](mailto:mdramire@arizona.edu)
1177 E 4^th^ St, Shantz 429, Tucson, AZ 85719, USA.

**Interview questions for Summer 2024 (SU24) follow-up interviews**

Introduction: Thank you for your time today. This interview is about your experience in the STEAM in Action training you completed in Spring/Summer 2024.

**Section 1. Environmental Health Knowledge**

1. Recall the term, environmental justice. Tell me what it means to you.
2. Now, talk to me about possible connections. Is there a connection between environmental justice and health? If so, why? If not, why not?
   - Prompt – have them elaborate on their response, “Oh tell me more about that!”…

**Section 2. Training and skill/knowledge acquisition**

1. What environmental science topics do you remember learning?
   - Prompt – have them elaborate on the topic, “Oh tell me more about that!”…
2. What do you remember about the soil and air monitoring activities?
3. What was the experience like collecting samples and completing the experiment?
4. What do you remember about the photovoice activity?
5. What was the photovoice experience like? Would you recommend it? Why or why not?
6. How, if it is applicable, have you used/applied what you learned in the summer training to other areas of your life – home, school, work, etc.?

**Section 3. Communication**

1. Why did you decide (or not) to pursue a position on the advisory board?
2. Have you shared what you learned with others in your community – friends, parents, siblings, coworkers, etc.? If so, in what ways have you shared what you learned? What are the kinds of things you shared?

**Section 4. Participant Satisfaction**

1. What was your favorite aspect of the summer training? What was your least favorite part? What are your suggestions to improve the summer training experience?

**Section 5. Efficacy**

1. What do you think are the most effective ways to address issues in your community?
2. In what ways can **YOU** make a difference in your community?
3. Do you feel that the summer training provided skills/resources/information to help you make a difference in your community? If so, what was most helpful/useful? If not, what would be helpful?
4. What do you see as challenges to making a difference in your community?
