# Supplementary material for: Building youth power and environmental health literacy with environmental justice communities in rural Arizona
Source: Front Public Health. 2026 May 12;14:1733720. doi: 10.3389/fpubh.2026.1733720 (PMC13201490; doi:10.3389/fpubh.2026.1733720)
Supplement: Supplementary file 6 [file Data_Sheet_6.docx]

Supplemental Figures for Building youth power and environmental health literacy with environmental justice communities in rural Arizona

Kunal Palawat^1^, William Borkan^1^, Sanlyn Buxner^2^, Isabella M. Castañeda^3^, Sallie Choi^3^, Ted Choi^3^, God’sgift N. Chukwuonye^1^, Melissa Jaquez^1^, Miriam Jones^1^, Anastasia Mariscal^3^, Miracle Martinez^1,4^, Spencer T. McBride^3^, Carol Newbauer^1^, Caleb Ochoa^3^, Benjamin Quesada^3^, Maricela Quesada^3^, Raquel N. Quesada^3^, Iliana A. Samorano^1^, Felix L. Vincent^3^, Abigail Zettlemoyer^1^, Mónica D. Ramírez-Andreotta^1,5*^

Affiliations

^1^Department of Environmental Science, College of Agriculture, Life, and Environmental Sciences, University of Arizona, Tucson, AZ, USA

^2^College of Education, University of Arizona, Tucson, AZ, USA

^3^Youth Advisory Board, “STEAM in Action”, Arizona, USA

^4^Regenerating Sonora, Inc., Superior, AZ, USA

^5^Mel and Enid Zuckerman College of Public Health, University of Arizona, Tucson, AZ, USA

All authors except for first and last are listed alphabetically.

*Corresponding author: Dr. Mónica D. Ramírez-Andreotta; [mdramire@arizona.edu](mailto:mdramire@arizona.edu)
1177 E 4^th^ St, Shantz 429, Tucson, AZ 85719, USA.


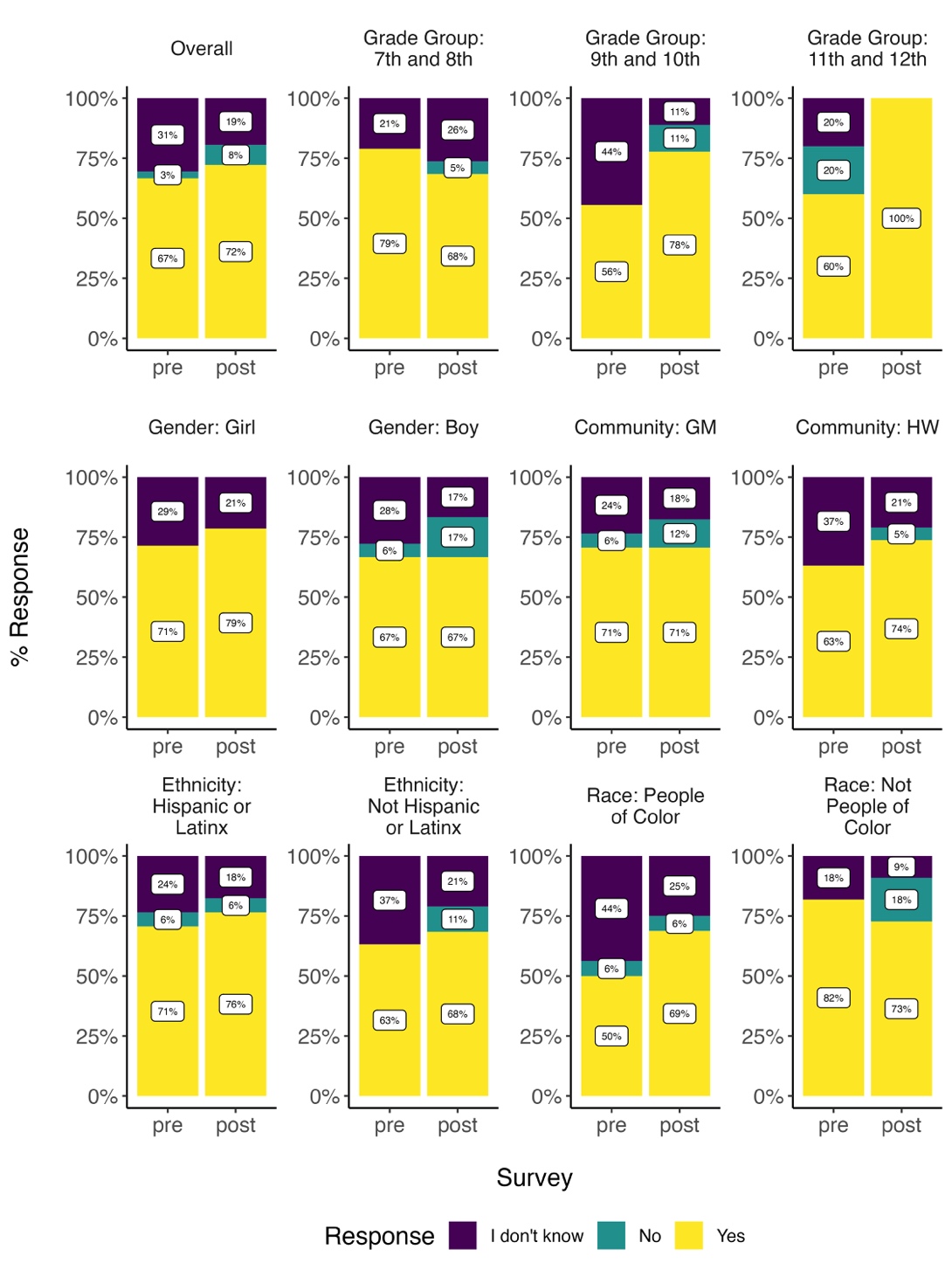


S Figure 1. Bar plots summarizing pre and post survey results for youth responding to the question, “Do you think that there are environmental problems in your community?”. Results demonstrate environmental health literacy knowledge and awareness. HW stands for the Hayden training and GM stands for the Globe training.


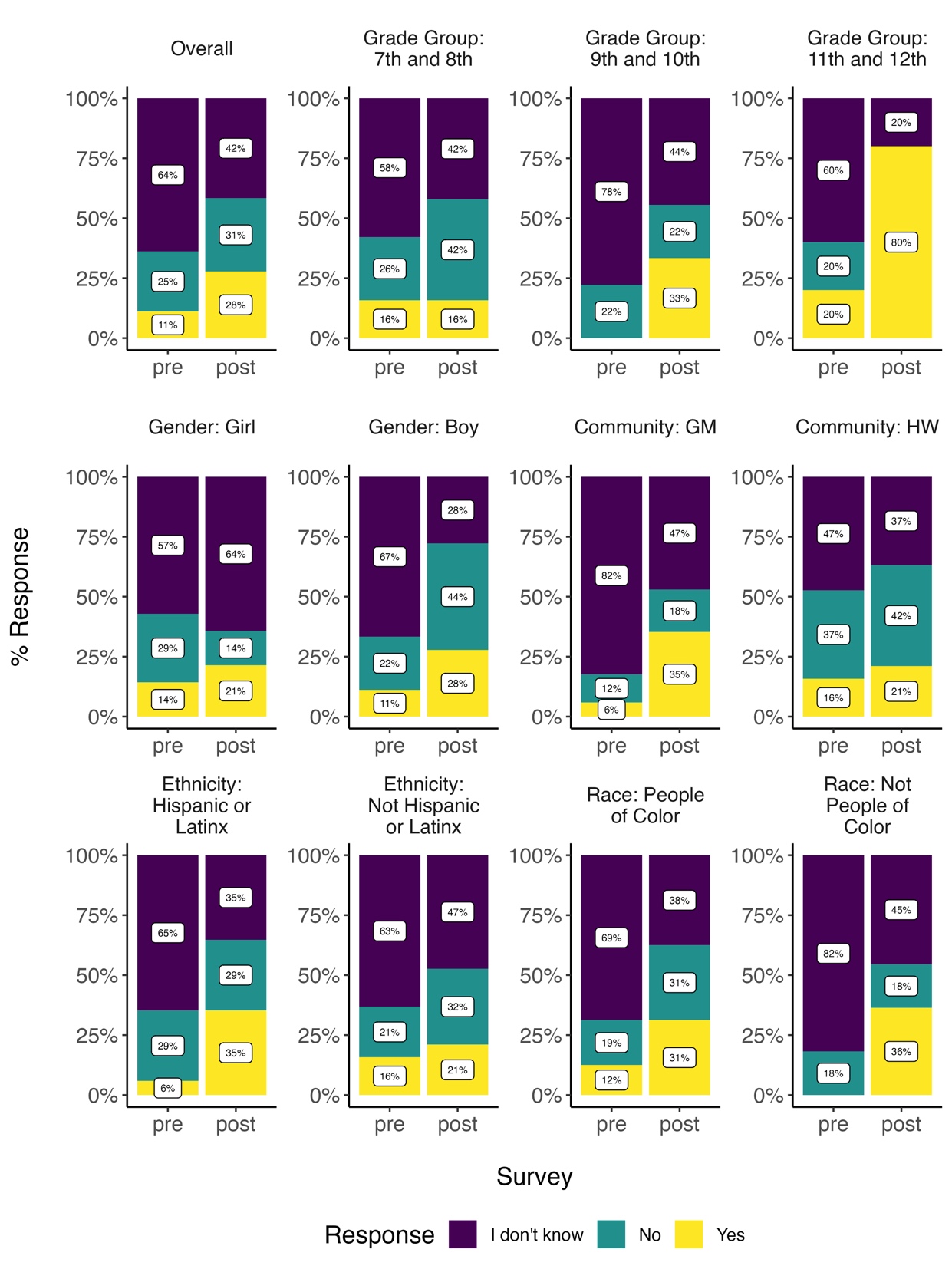


S Figure 2. Bar plots summarizing pre and post survey results for youth responding to the question, “Do you think that there are justice or injustice problems in your community?”. Results demonstrate environmental health literacy knowledge and awareness. HW stands for the Hayden training and GM stands for the Globe training.


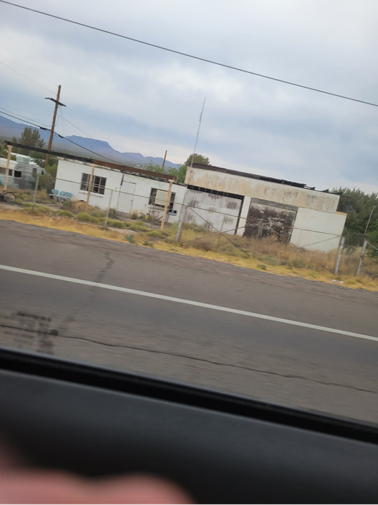


(A)

(D)

(C) Image removed to maintain anonymity.

(B) Image removed to maintain anonymity.


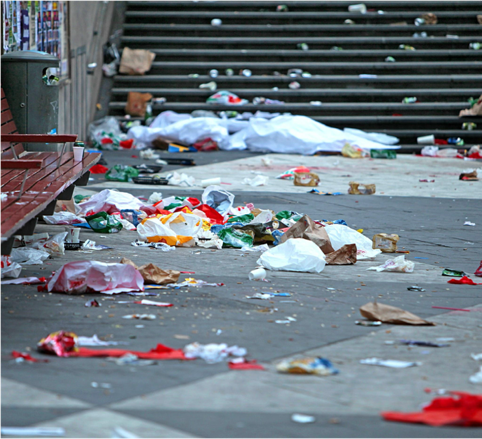

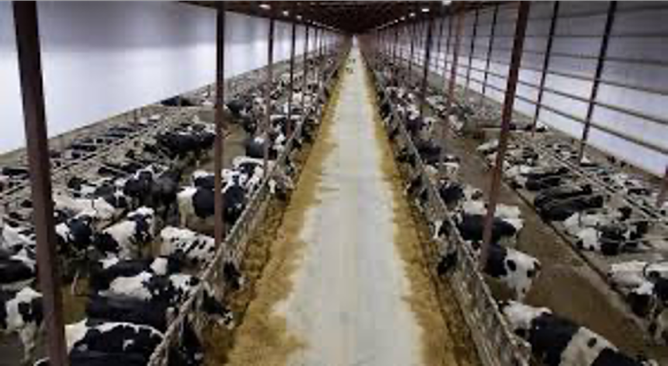


(E)

S Figure 3. Youth photovoice highlights. Captions read: (A) “I took this picture because I think we should spend more time on refurbishing buildings back to their old use and look. Or we can refurbish them and use them for something new.” (B) “Our gym needs better air conditioning. If you play sports you know you are constantly in the gym but most people don’t go cause of how hot it gets. Some people don’t go to games because of how hot it gets.” (C) “The vibe is all about connection with each other, nature, and the environment around us. You can feel the warmth of togetherness and how we’re all just soaking in the surroundings. It’s like we’re not just sitting on a bench talking, but we’re really embracing the nature around us. The way we’re all gathered there shows a real appreciation of the environment we’re in. It’s like we’re [part] of a scene, not just observers.” (D) “The streets are full of trash.” (E) “Believe it or not cows are some of the biggest contributors to climate change, and not in a good way. Cows emit Methane gas from their farts…” See more at <https://explore.steamaction.arizona.edu/photovoice>.


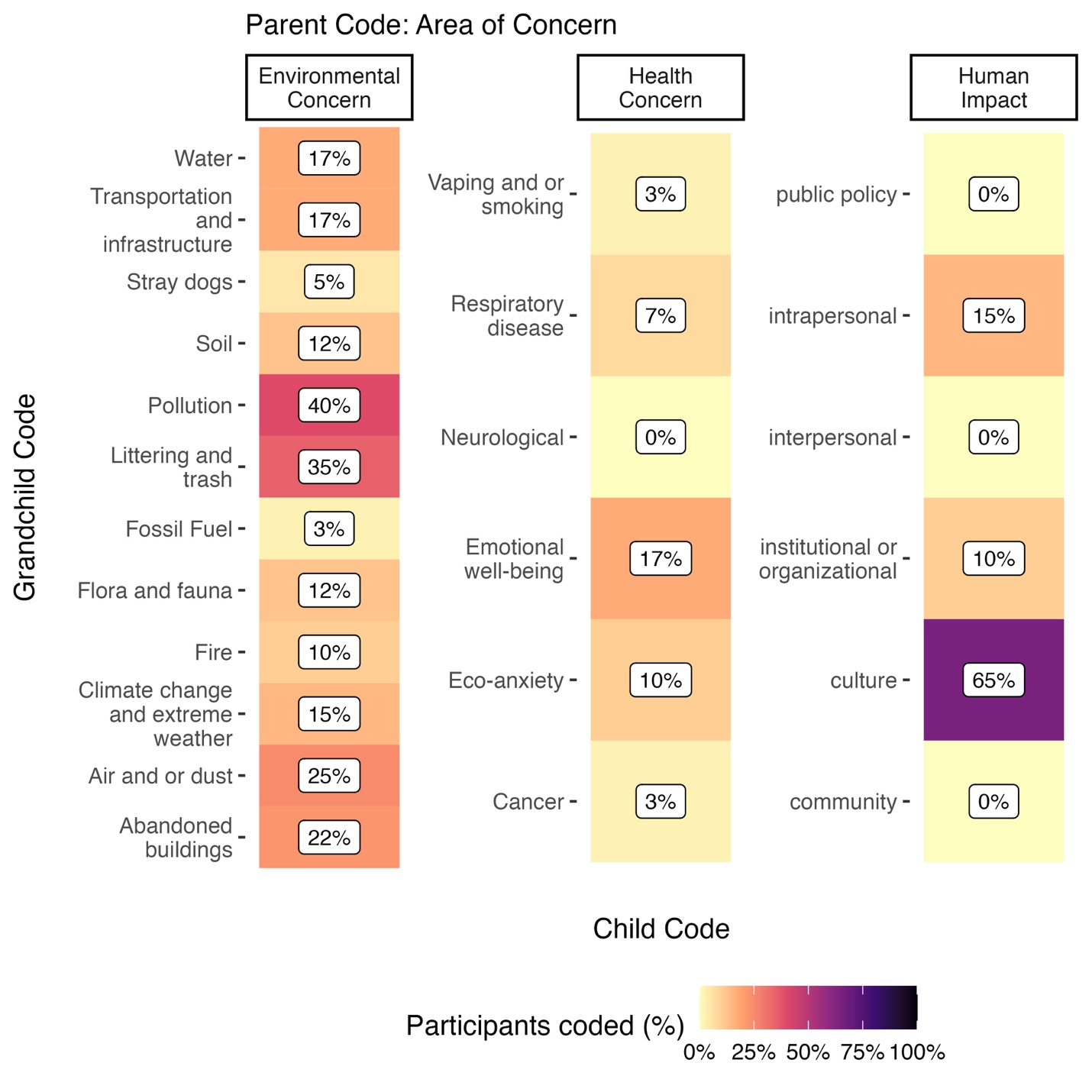


S Figure 4. Bar plot showing the percentages of focus group youth saying something coded to (A) environmental concern indicating environmental health literacy knowledge and awareness (B) health concern indicating environmental health literacy knowledge and awareness and (C) human impact, indicating environmental health literacy knowledge and awareness and community change.


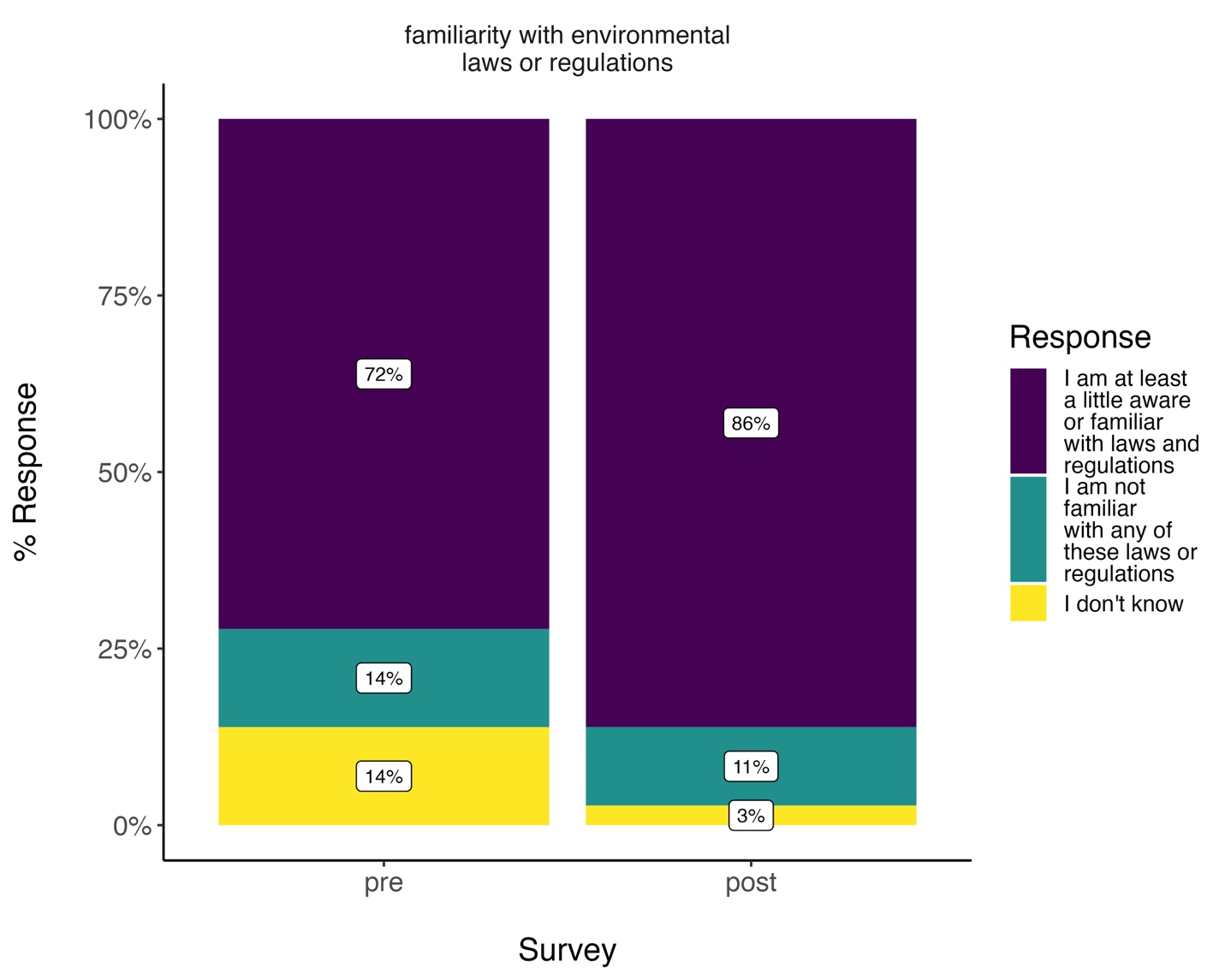


S Figure 5. Bar plot summarizing pre and post survey results for 36 youth who took both the pre and post surveys. Plots show responses asking about familiarity with environmental laws or regulations, demonstrating environmental health literacy knowledge and awareness.


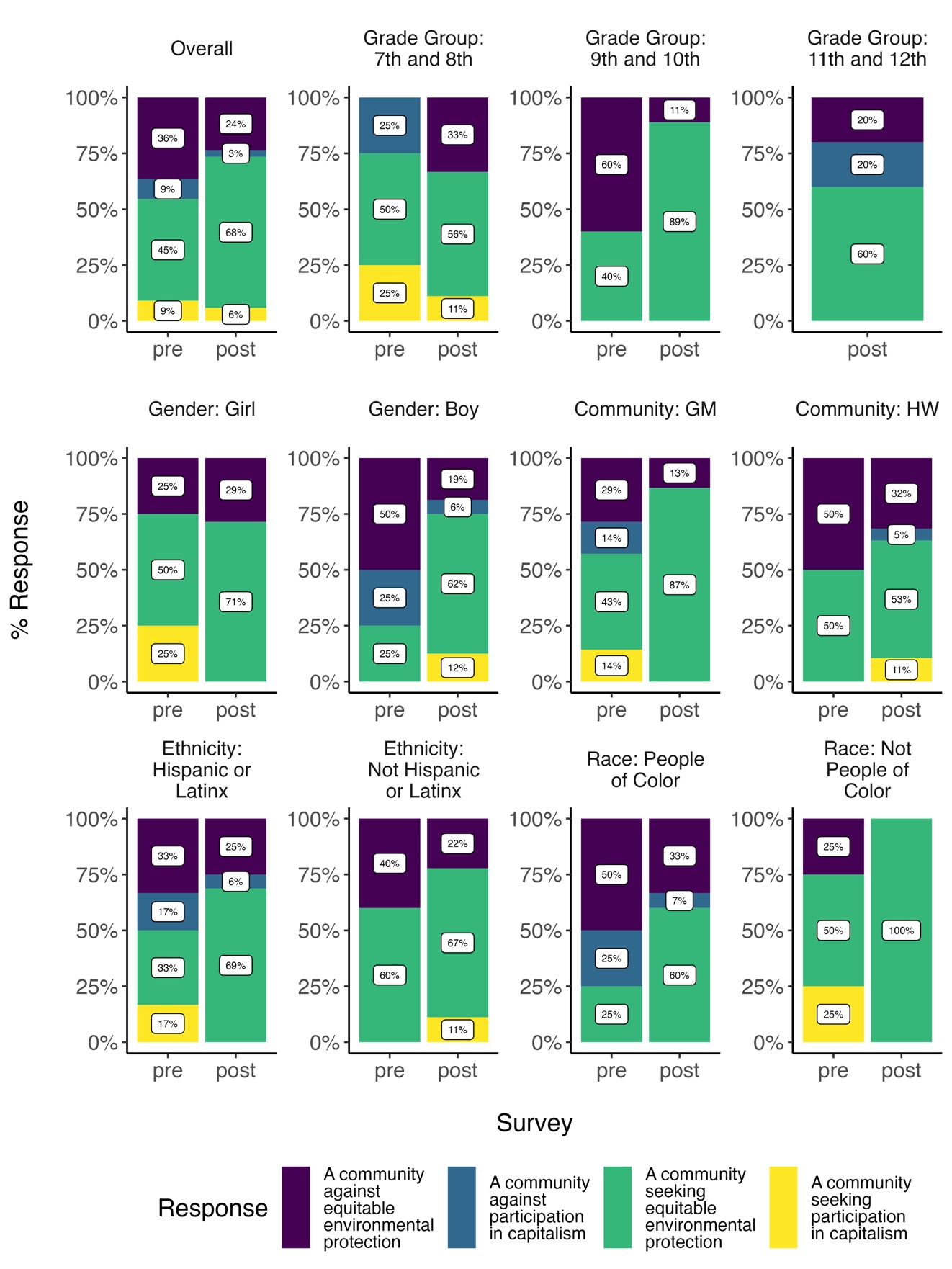


S Figure 6. Bar plot summarizing pre and post survey results for youth who took both the pre and post surveys. Plots show responses to defining the term, “environmental justice”, demonstrating environmental health literacy knowledge and awareness.


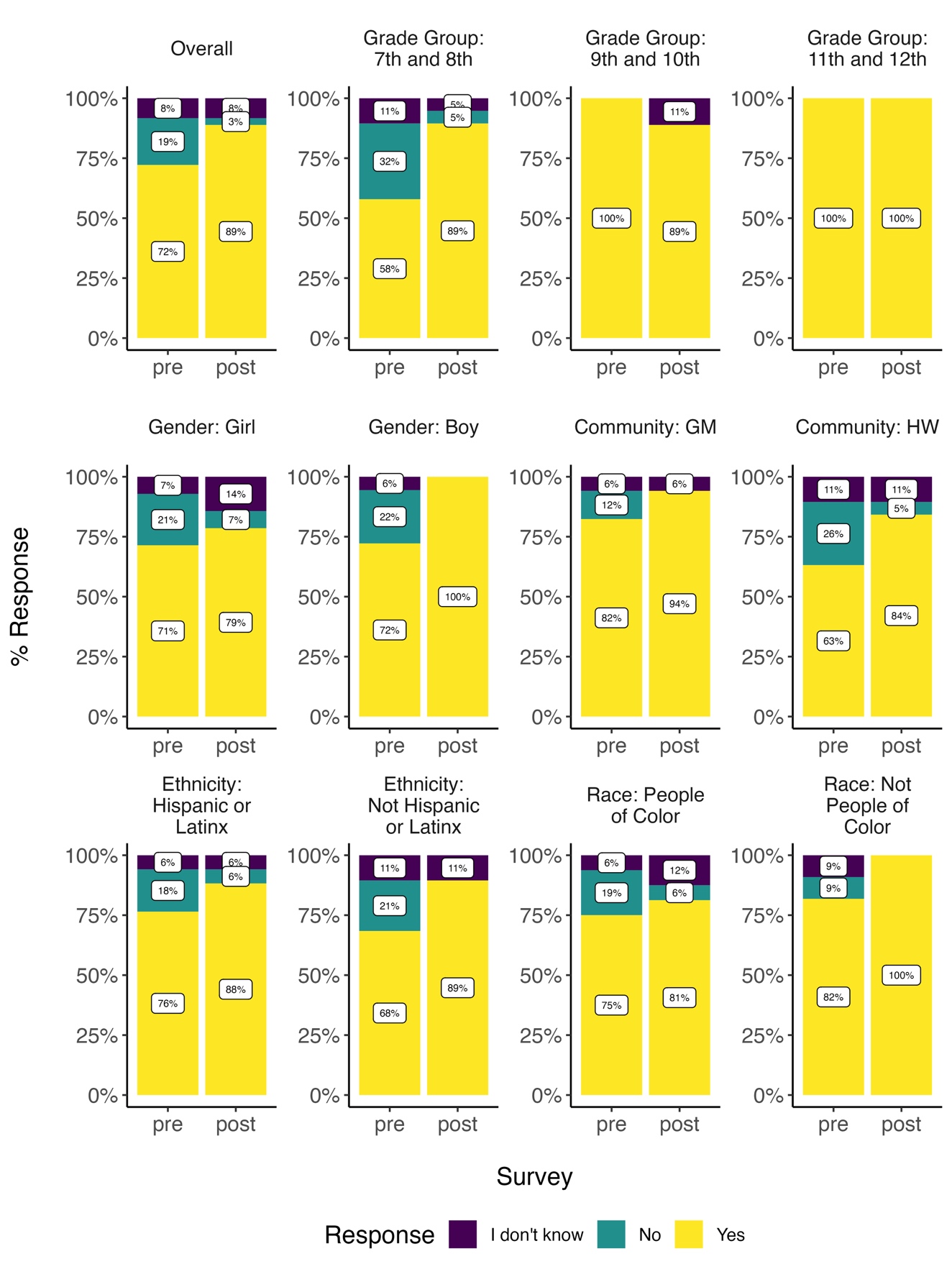


S Figure 7. Bar plot summarizing pre and post survey results for youth who took both the pre and post surveys. Plots show responses to familiarity with the term, “public health issue”, demonstrating environmental health literacy knowledge and awareness.


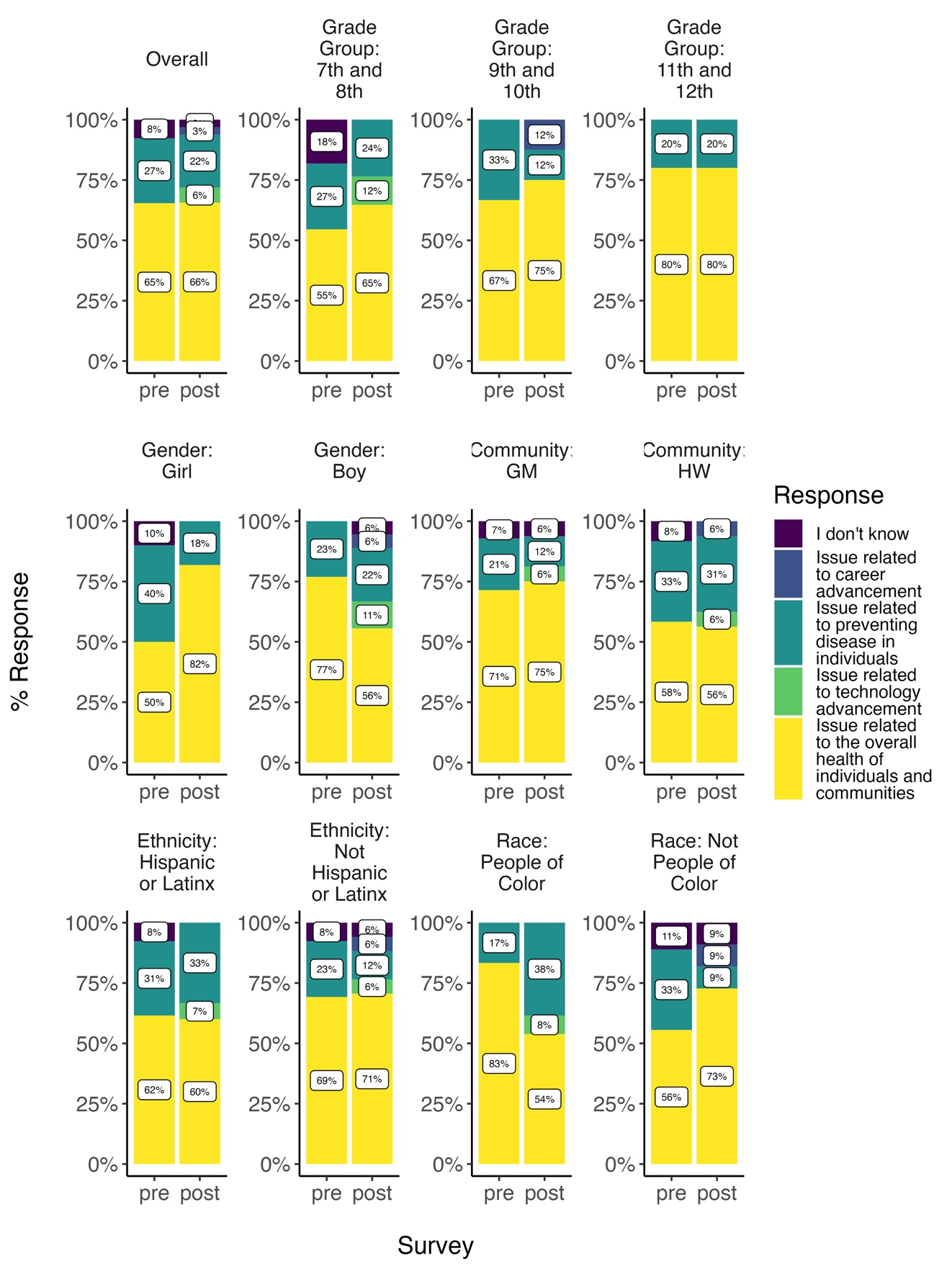


S Figure 8. Bar plot summarizing pre and post survey results for youth who took both the pre and post surveys. Plots show responses to defining the term, “public health issue”, demonstrating environmental health literacy knowledge and awareness.


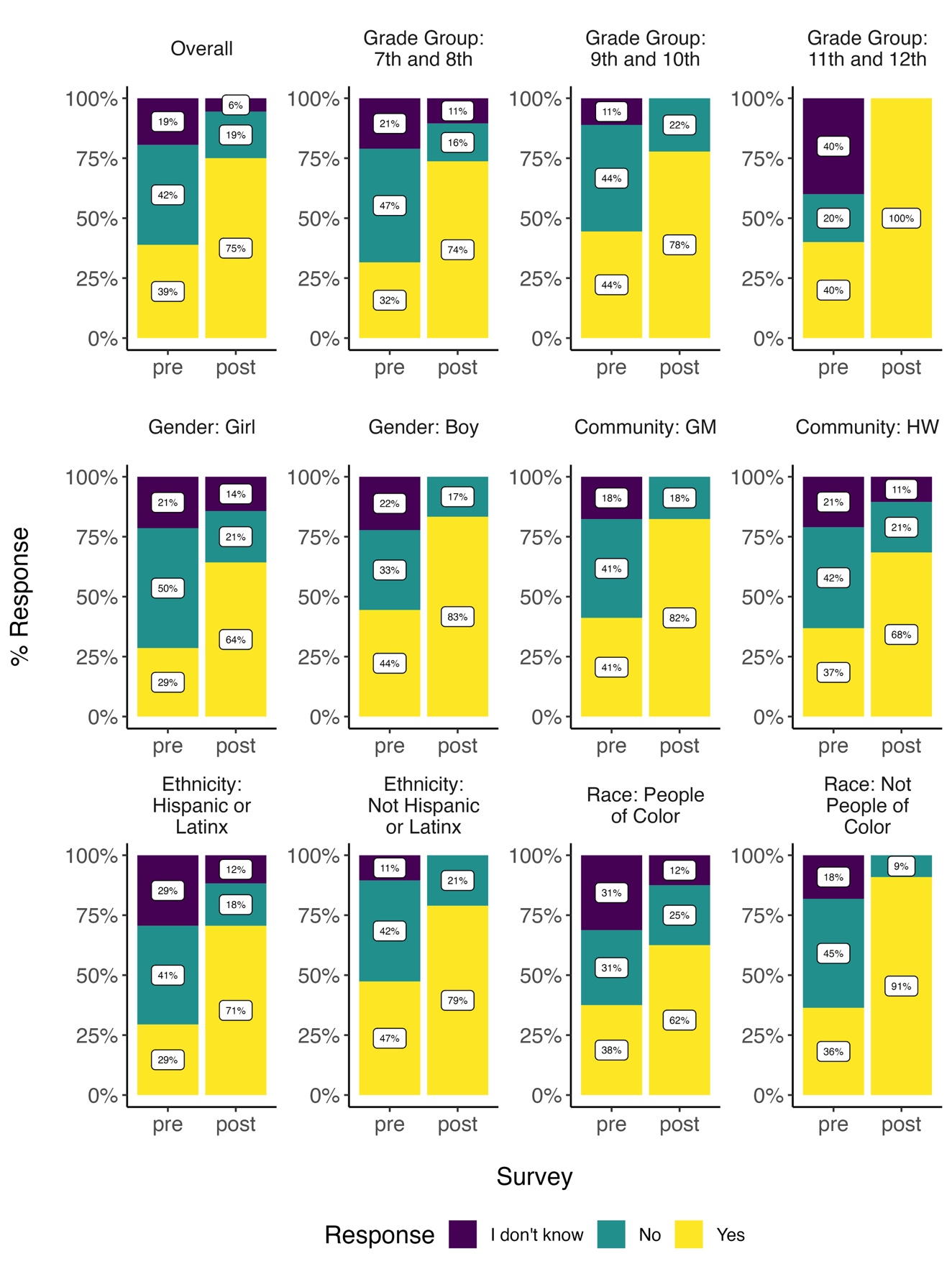


S Figure 9. Bar plot summarizing pre and post survey results for youth who took both the pre and post surveys. Plots show responses to familiarity with the term, “environmental monitoring”, demonstrating environmental health literacy knowledge and awareness.


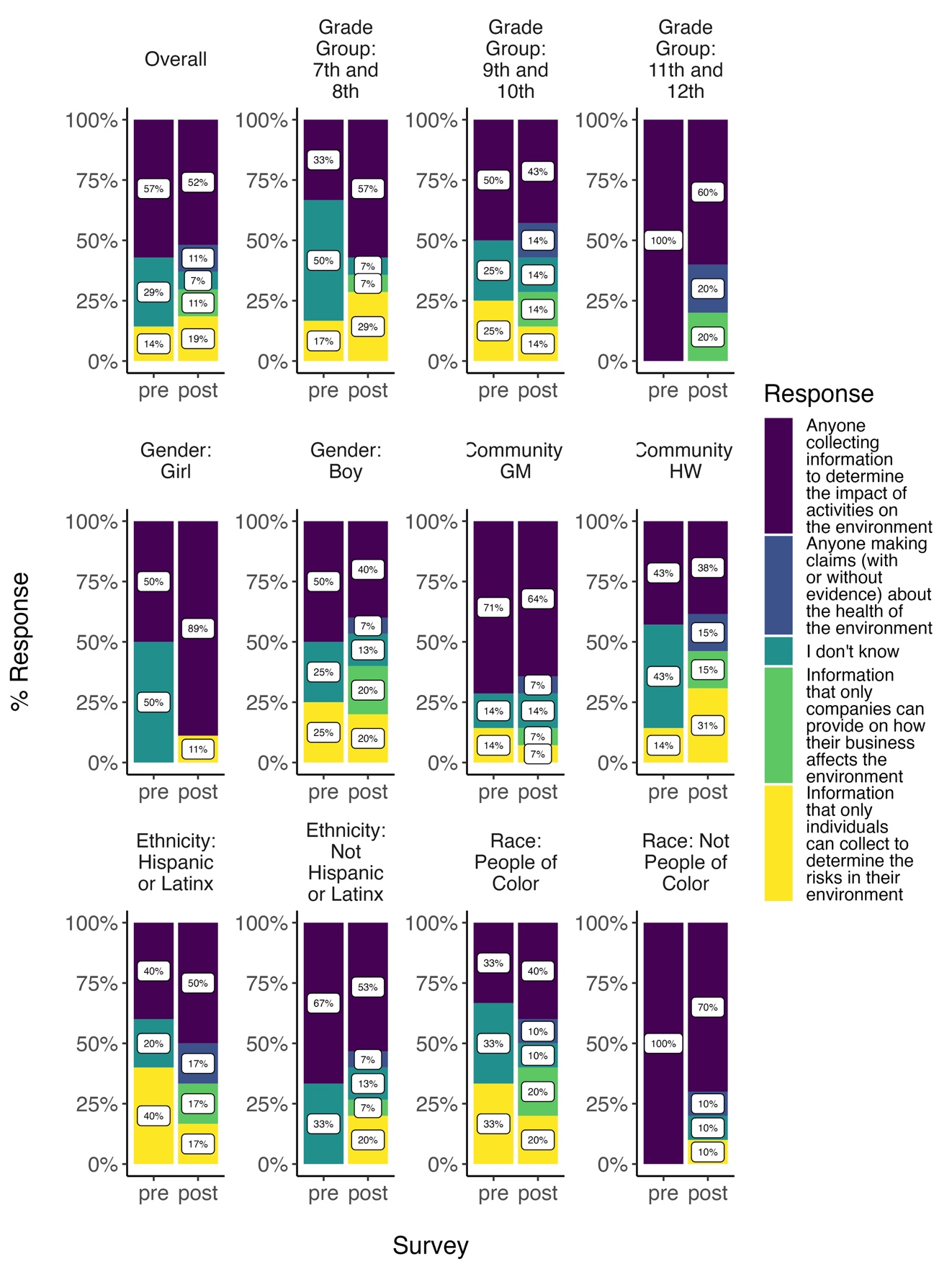


S Figure 10. Bar plot summarizing pre and post survey results for youth who took both the pre and post surveys. Plots show responses to defining the term, “environmental monitoring”, demonstrating environmental health literacy knowledge and awareness.


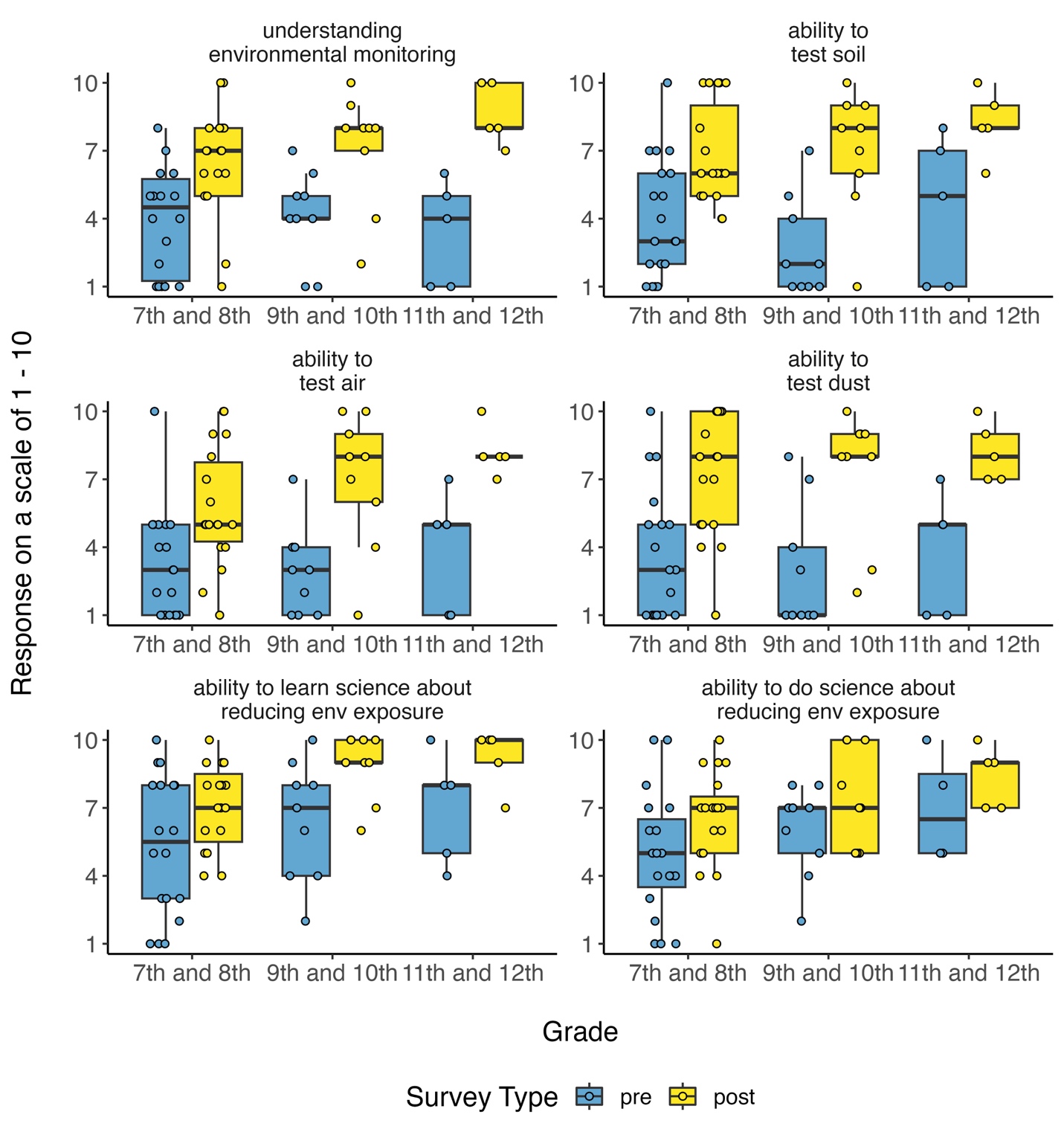


S Figure 11. Box plots showing the self-rated confidence in learning and doing science demonstrating environmental health literacy self-efficacy by grade group.


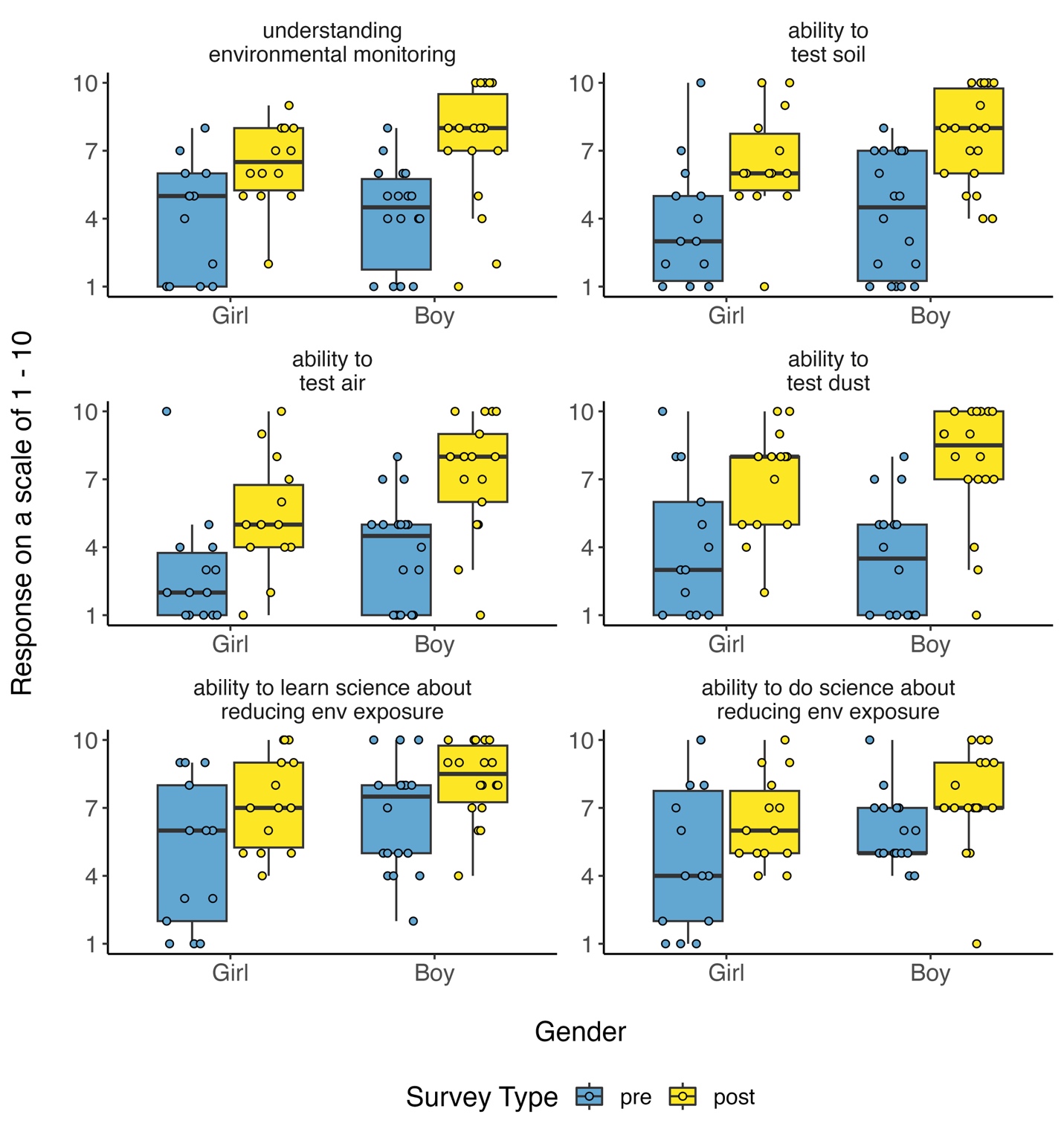


S Figure 12. Box plots showing the self-rated confidence in learning and doing science demonstrating environmental health literacy self-efficacy by gender.


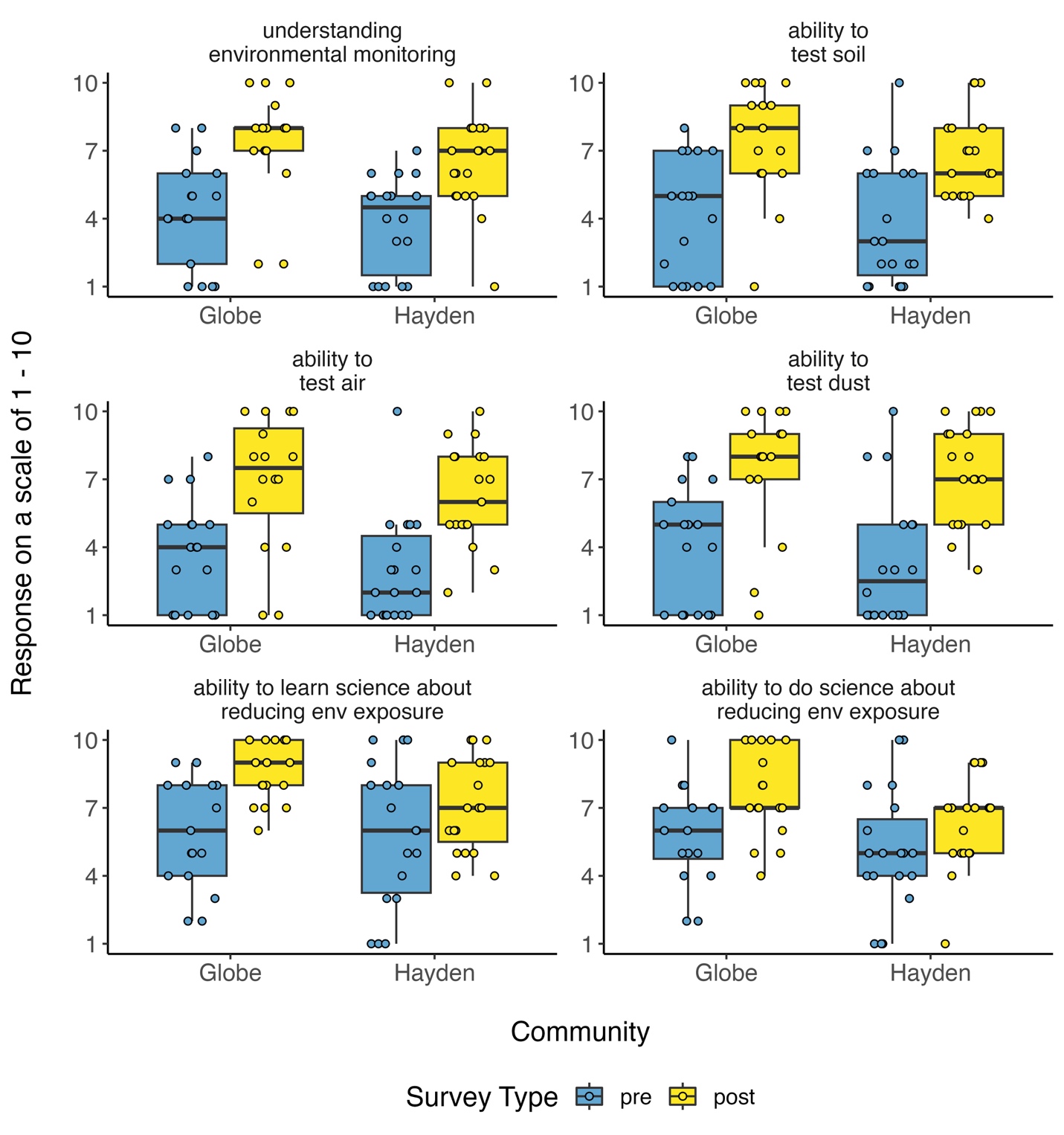


S Figure 13. Box plots showing the self-rated confidence in learning and doing science demonstrating environmental health literacy self-efficacy by community.


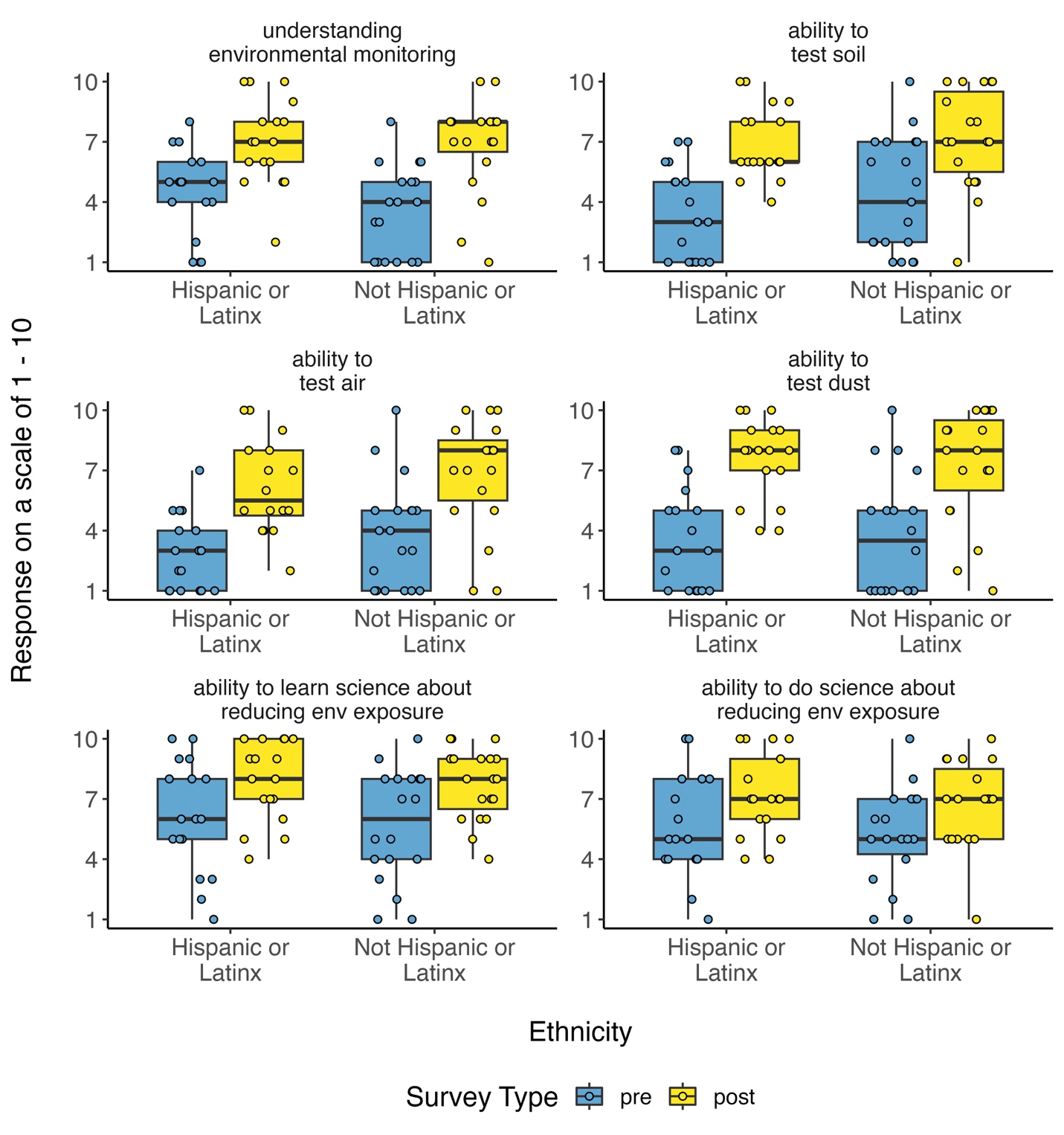


S Figure 14. Box plots showing the self-rated confidence in learning and doing science demonstrating environmental health literacy self-efficacy by ethnicity.


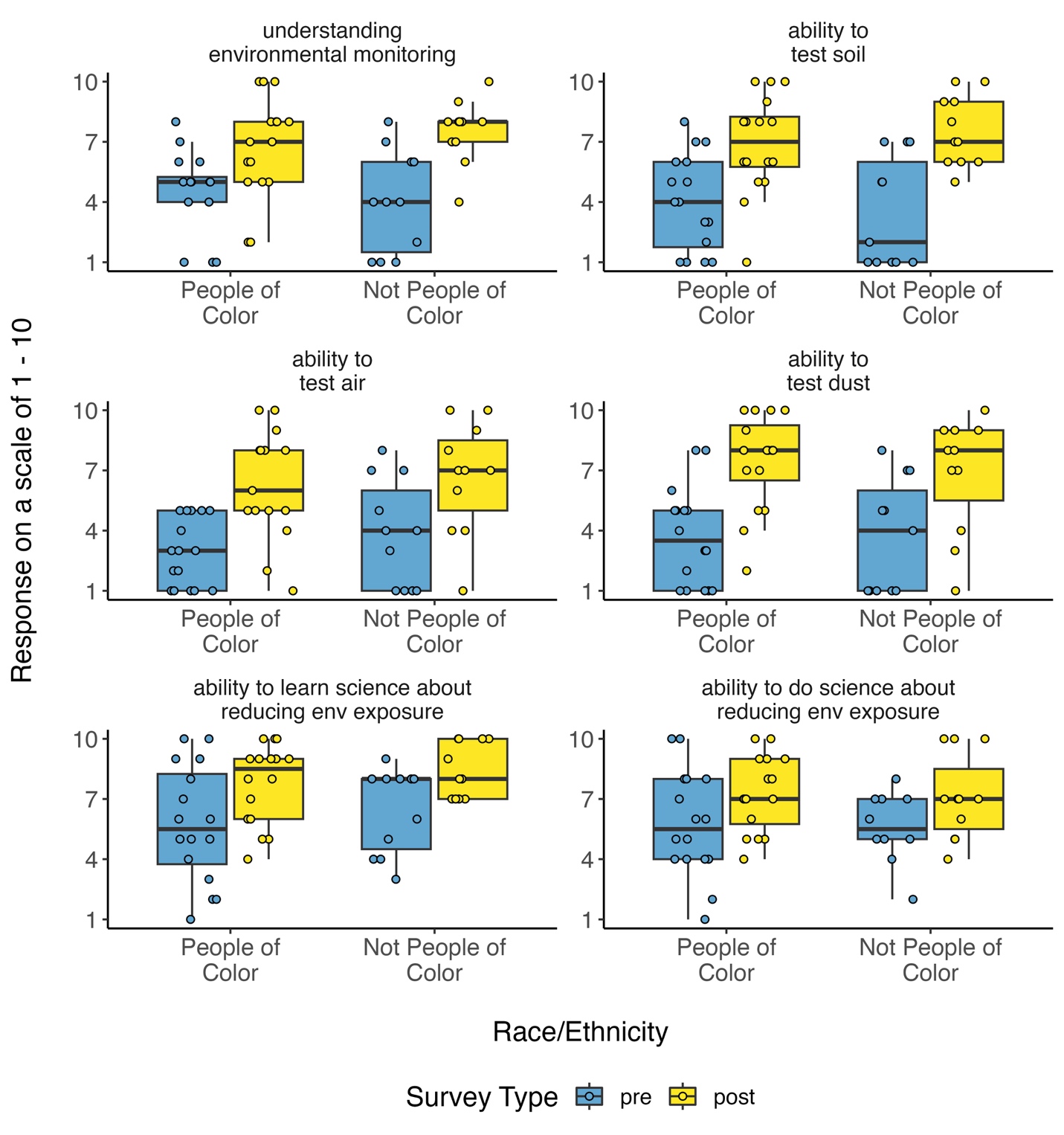


S Figure 15. Box plots showing the self-rated confidence in learning and doing science demonstrating environmental health literacy self-efficacy by race.


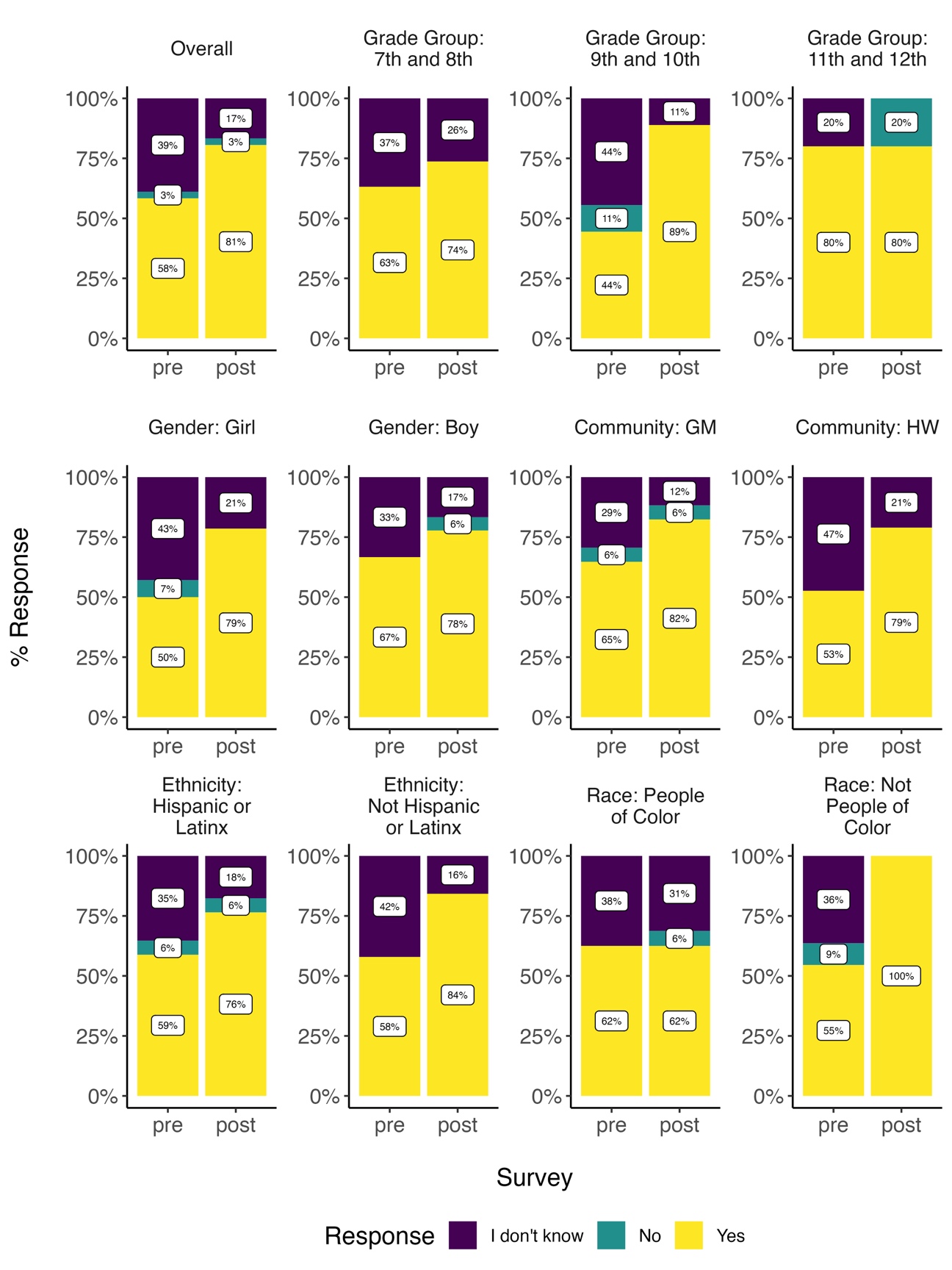


S Figure 16. Bar plot summarizing pre and post survey results for 36 youth who took both the pre and post surveys, perception of one’s community’s ability to make decisions social justice issues, demonstrating environmental health literacy self-efficacy and community change.


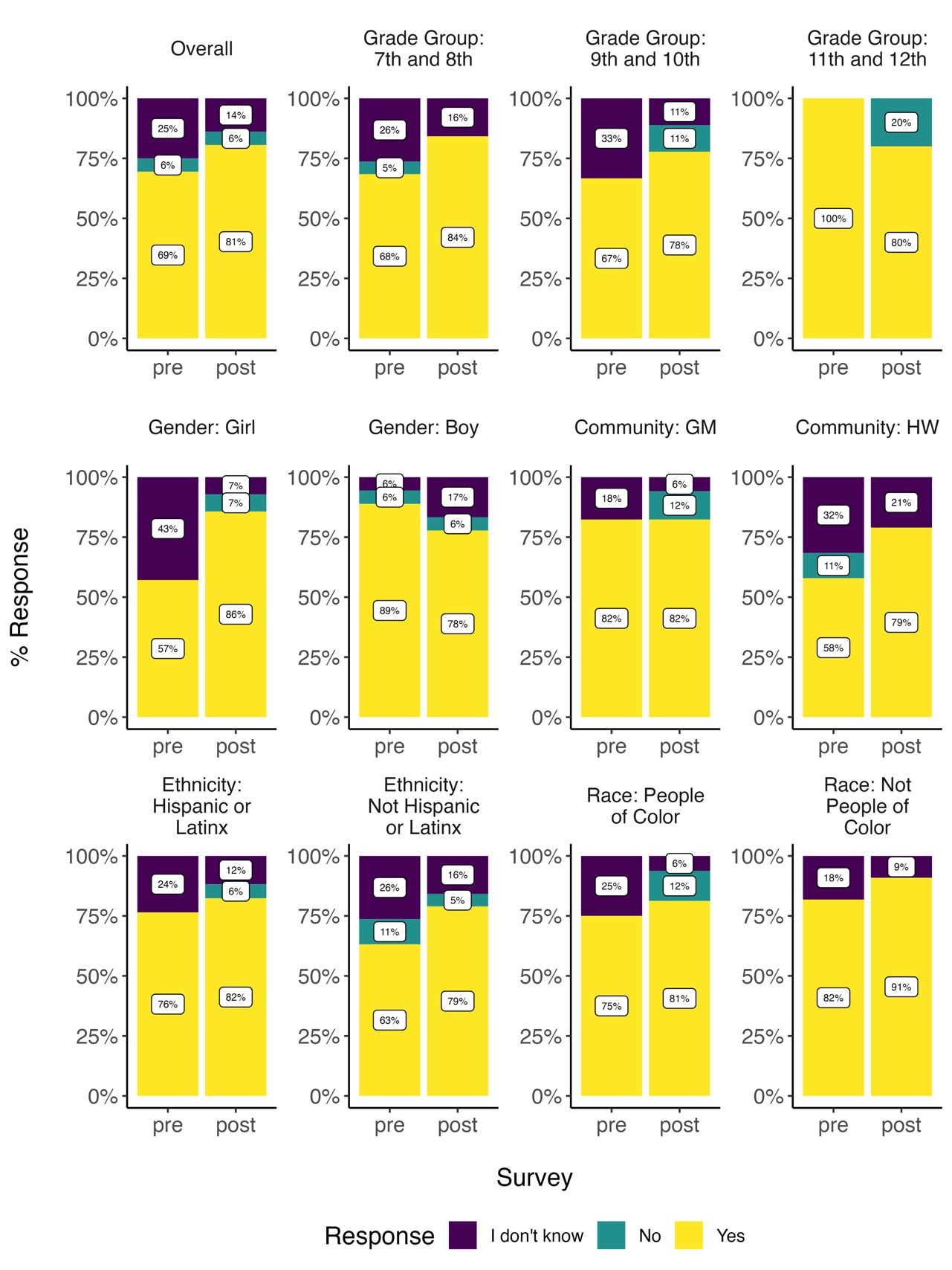


S Figure 17. Bar plot summarizing pre and post survey results for 36 youth who took both the pre and post surveys, perception of one’s community’s ability to make decisions about having a safe environment to live in, demonstrating environmental health literacy self-efficacy and community change.


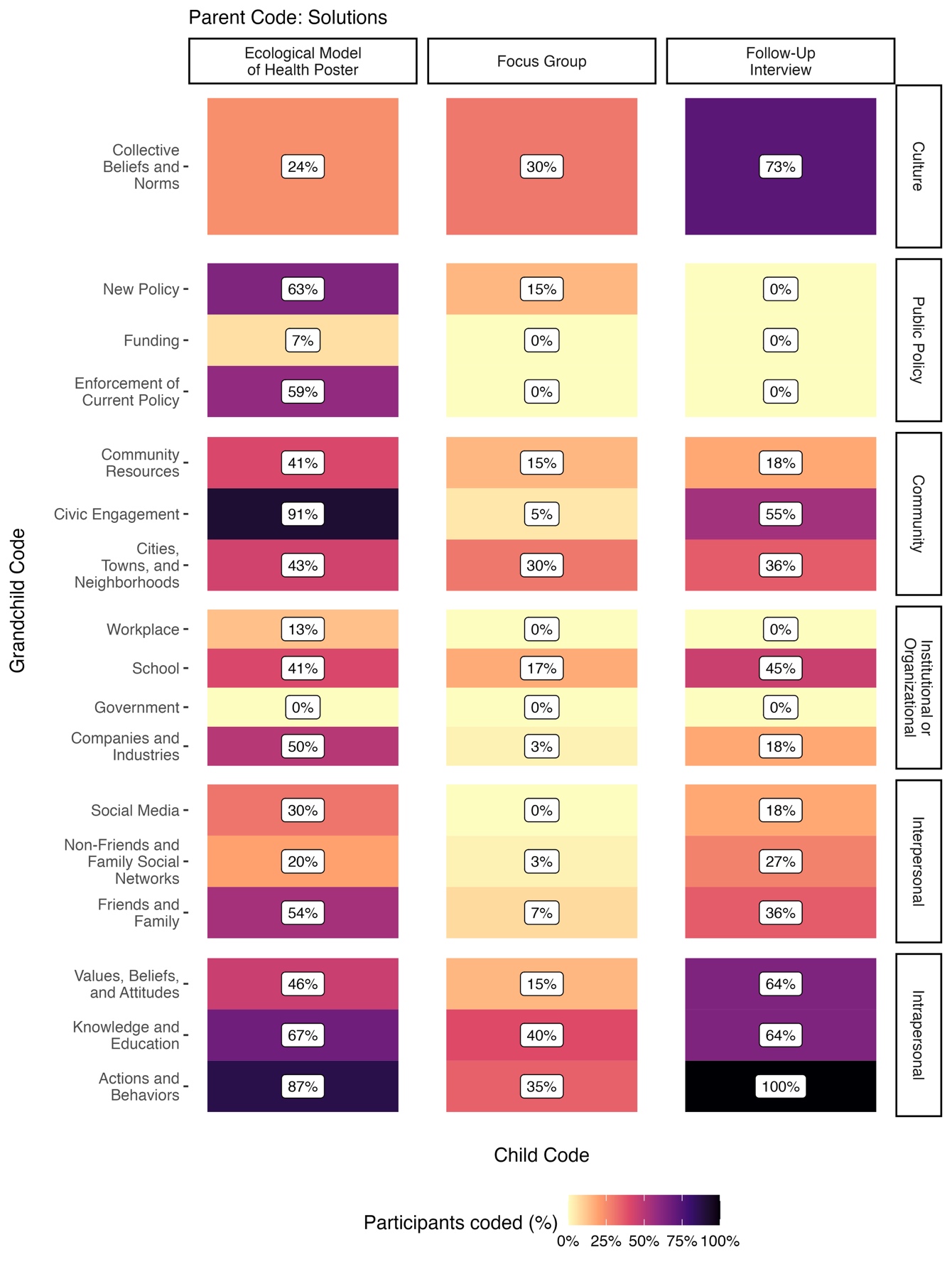


S Figure 18. Bar plot showing the percentages of youth saying something coded to solutions, demonstrating environmental health literacy knowledge and awareness, self-efficacy, and community change. Plots show data for ecological model of health posers (N = 46), focus group (N = 40), and follow up interviews (N=11).


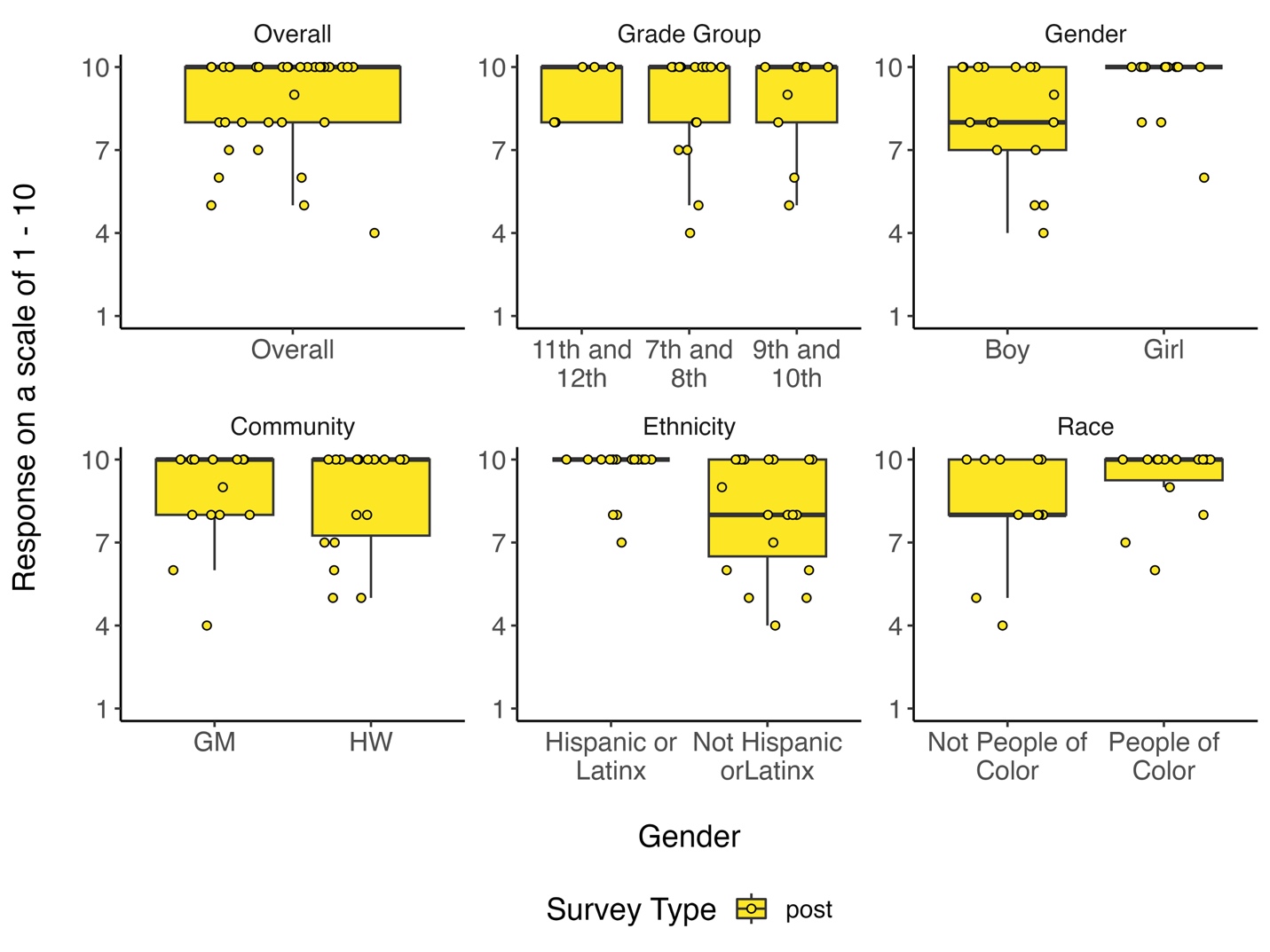


S Figure 19. Box plots showing the self-rated satisfaction with the STEAM in Action training.
